# Rapid generation of sequence-diverse terminator libraries and their parameterization using quantitative Term-Seq

**DOI:** 10.1093/synbio/ysz026

**Published:** 2019-10-29

**Authors:** Andrew J Hudson, Hans-Joachim Wieden

**Affiliations:** 1 Alberta RNA Research and Training Institute (ARRTI), University of Lethbridge, Lethbridge, Alberta, Canada; 2 Department of Biological Sciences, University of Lethbridge, Lethbridge, Alberta, Canada; 3 Department of Chemistry and Biochemistry, University of Lethbridge, Lethbridge, Alberta, Canada

**Keywords:** transcriptional regulatory elements, genetic circuit engineering, biological techniques, gene expression regulation, synthetic biology

## Abstract

Synthetic biology and the rational design and construction of biological devices require vast numbers of characterized biological parts, as well as reliable design tools to build increasingly complex, multigene architectures. Design principles for intrinsic terminators have been established; however, additional sequence-structure studies are needed to refine parameters for termination-based genetic devices. We report a rapid single-pot method to generate libraries of thousands of randomized bidirectional intrinsic terminators and a modified quantitative Term-Seq (qTerm-Seq) method to simultaneously identify terminator sequences and measure their termination efficiencies (TEs). Using qTerm-Seq, we characterize hundreds of additional strong terminators (TE > 90%) with some terminators reducing transcription read-through by up to 1000-fold in *Escherichia coli*. Our terminator library and qTerm-Seq pipeline constitute a flexible platform enabling identification of terminator parts that can achieve transcription termination not only over a desired range but also to investigate their sequence-structure features, including for specific genetic and application contexts beyond the common *in vivo* systems such as *E. coli*.

## 1. Introduction

Forward engineering of genetic devices to carry out useful functions is at the very core of synthetic biology. However, achieving predictable control of gene expression requires a detailed understanding of relevant biological processes and the availability of a sufficient number of characterized genetic parts for gene construction (1). Genetic parts libraries have been developed mostly for bacteria (especially *Escherichia coli*) and they have provided genetic engineers with repertoires to modulate gene expression at the transcriptional (2–5), post-transcriptional (6, 7) and translational levels (8–10). Experimental characterization of single or collections of natural or synthetic parts has also facilitated predictive models to assist with synthetic cistron construction (5, 11, 12).

Engineered genetic circuits consisting of one or a few genes have been successfully applied to create a variety of genetic devices such as: oscillators (13), toggle switches (14) and logic gates (15). However, *de novo* engineering of more complex, multigene synthetic devices are still limited by an incomplete understanding of fundamental mechanisms of gene expression and/or accurate modeling of complex cellular environments and gene interactions. Indeed, standardizing biological parts with predictable properties remains challenged by the finding that part performance may differ drastically in different DNA (or RNA transcript) sequence contexts (16). Development of high-throughput methods that characterize the performance of large collections of genetic parts under specific physiological conditions and genetic contexts are, therefore, needed to refine genetic design principles and afford finer control over gene expression.

Transcription termination is a fundamental step of gene expression in all organisms and serves to prevent unintended transcription of flanking gene sequences, to define RNA transcript 3′ ends and to recycle RNA polymerase for subsequent rounds of transcription (17). In bacteria, transcriptional terminators are found near the end of operons where they modulate transcription of downstream genes and, in some cases, make regulatory decisions in response to changing cellular conditions (e.g. small molecule recognition by terminator/anti-terminator riboswitches) (18). Compared with other basic biological parts (e.g. transcription promoters and ribosome binding sites), transcription terminators have been less explored as tools to control gene expression; however, their utility as synthetic gene expression regulators is exemplified by their repurposing as adjustable high-pass and low-pass biological filters (19) and their integration into functional synthetic riboswitches (20). Moreover, recent high-throughput RNAseq strategies such as Term-Seq (21) have facilitated the discovery of novel riboregulators which provide additional gene expression regulation tools available to synthetic biologists and provide additional mechanistic insights into factor-dependent and intrinsic termination mechanisms (21, 22).

The mechanism and sequence features affecting intrinsic termination efficiency (TE) have been extensively characterized (17). In *E. coli*, intrinsic terminators minimally consist of a G-C-rich hairpin (T_hp_) that is immediately followed by a 7–9 nucleotide poly(U) tract (23). Intrinsic termination begins with stalling of RNA polymerase on the poly(U) tract due to the especially weak rU-dA hybrid and this creates a kinetic window for T_hp_ formation in the nascent RNA (24). Invasion of the T_hp_ into the RNA exit channel of RNAP then results in shearing of the rU-dA hybrid (25, 26) or hyper-translocation of RNAP (25, 27) with concomitant structural changes in RNAP that promote transcription elongation complex (TEC) dissociation (17). Stronger base pairing of the T_hp_ and a perfect, extended poly(U) tract are associated with increased TE (12) and the strength of the four closing base pairs of the T_hp_ and first three uridines of the poly(U) tract are particularly conserved features of strong intrinsic terminators (12, 23). Some intrinsic terminators also possess a poly(A) tract upstream of the T_hp_ and this feature is generally associated with increased TE and can enable terminators to act in both forward and reverse directions—so-called bidirectional terminators (17).

Intrinsic terminator libraries are valuable tools for examining RNA/DNA sequence features that affect TE and for identifying sequence-diverse intrinsic terminators with specified efficiencies for genetic circuit design (12); however, to the best of our knowledge, no current method can generate bacterial transcriptional terminator libraries with randomized hairpin sequences without requiring many oligonucleotides (i.e. one or two oligos per terminator) or relatively expensive massively parallel DNA synthesis strategies. Development of inexpensive and straightforward methods would facilitate high-throughput studies to further dissect RNA and DNA sequences and/or structures affecting intrinsic termination and provide a greater collection of characterized terminators for genetic engineering. Here, we describe a novel workflow to generate thousands of bacterial intrinsic terminators with flexible design parameters using only two DNA oligonucleotides and commonplace molecular biology enzymes and equipment. We also apply a variation of the high-throughput terminator RNA-sequencing methodology Term-Seq (21) to examine synthetic terminator performance *in vivo* under a variety of conditions. Applying these methods, we characterize hundreds of additional strong intrinsic terminators in forward and reverse orientations that can be used for engineering bacterial cistrons. These methods provide a foundation for larger-scale studies to dissect sequence-structure features influencing transcription termination and to identify synthetic riboregulators that achieve a desired TE and/or respond to changing cellular conditions and chassis.

## 2. Materials and methods

### 2.1 Terminator library construction

Terminator libraries were assembled in four steps consisting of: (i) oligo annealing, (ii) oligo extension, (iii) oligo ligation and (iv) polymerase chain reaction (PCR; Supplementary Note S1). All oligonucleotides for the study were synthesized by Integrated DNA Technologies and are detailed in Supplementary Table S1.

- *Oligo annealing* One hundred picomoles of 5ʹ half (oTerm13) and 5ʹ-end monophosphorylated 3ʹ half (oTerm14) oligonucleotides were mixed in 100 µl of ribonuclease-free ultrapure water (MilliQ^®^) and heated in a thermocycler to 95°C for 2 min, followed by slowly cooling at a rate of 2°C per minute until the sample reached 21°C. Samples were then placed on ice.
- *Oligo extension* Annealed oligos were extended by T4 DNA polymerase for 10 min at 21°C in 20 µl reactions containing 10 pmol of annealed oligos, 1× NEBuffer 2.1 (New England Biolabs, NEB), 250 nmol each dNTP and 3 units of T4 DNA polymerase (NEB). Extended oligo complexes were then extracted via phenol-chloroform at 21°C, ethanol precipitated and suspended in 15 µl of nuclease-free MilliQ^®^ ddH_2_O.
- *Oligo ligation* Oligo 5ʹ and 3ʹ halves were ligated in 20 µl reactions containing 10 pmol annealed and extended oligos, 1× T4 DNA ligase buffer containing rATP (NEB) and 10 U T4 DNA ligase (NEB). Ligations were incubated for 20 min at 16°C and then heat-inactivated at 65°C for 15 min.
- *Polymerase chain reaction* One microliter of ligated oligo reaction was used as template in 100 µl PCR reactions containing 1× ThermoPol Buffer (Thermo Scientific), 200 µM dNTPs, 10 pmol each forward (oTerm16) and reverse (oTerm17) primers and 0.2 U Phusion DNA polymerase (Thermo Scientific). PCR was carried out with an initial denaturation step at 98°C for 30 s, 25 cycles consisting of 98°C for 10 s, 55°C for 10 s and 72°C for 20 s, and a final extension step at 72°C for 5 min. PCR products were verified on 8% native polyacrylamide gels, blunt-end cloned into the pJET1.2 (Thermo Scientific) using manufacturer’s protocols and fifty clones were subjected to Sanger sequencing to evaluate library quality and sequence complexity.

### 2.2 Dual fluorescence reporter and library cloning

The pBeRG reporter plasmid was created by cloning a synthetic gBlock cassette (Integrated DNA Technologies) containing an *E. coli* codon-optimized, N-terminally FLAG-tagged monomeric blue-exciting red fluorescent protein (mBeRFP) (28) coding sequence complete with a strong 5ʹ Shine-Dalgarno sequence and 3ʹ flanking BioBrick^®^ prefix and suffix sequences at the EcoRI and NdeI restriction sites upstream of the enhanced green fluorescent protein (eGFP) gene of the host plasmid pBbE6a (29) (see Supplementary Figure S1 for annotated plasmid sequence). mBeRFP and eGFP strongly excite at 488 nm but have widely separated emission peaks (610 and 510 nm, respectively) that minimize fluorescence resonance energy transfer and emission channel bleed-through. pBeRG was transformed into NEB5α *E. coli* chemically competent cells (NEB) and subsequently verified by Sanger sequencing. Terminator library PCR products were cloned nondirectionally into pBeRG at the two NotI sites of the BioBrick^®^ prefix and suffix sequences and 100 ng of ligated plasmid was transformed into NEB5α high-efficiency chemically competent *E. coli* cells. Several transformation products were pooled and plated on LB agar with 100 µg/ml ampicillin (LB_Amp100_). Transformation efficiency was estimated by plating 1% of transformation on separate LB_Amp100_ plates. The final terminator library (∼10 000 clones) was then suspended in 5 ml of LB_Amp100_ medium containing 20% glycerol, flash frozen in liquid nitrogen and stored at −80°C.

### 2.3 Flow cytometry and fluorescence-activated cell sorting experiments

Flow cytometry analysis of library clones (*n* = 180) as well as terminator control plasmid clones was performed by inoculating 5 ml LB_Amp100_ liquid medium with individual *E. coli* (NEB5α) colonies and growing at 37°C with shaking at 200 RPM for 16 h. Cultures were then used to inoculate fresh 5 ml LB_Amp100_ media, grown to mid-log phase (OD_600_ = 0.4) with shaking at 37°C and induced by addition of 1 mM IPTG. Fluorescent proteins were expressed overnight (16 h) to allow for maximal fluorescent protein maturation, prior to flow cytometry (see Supplementary Note S2 for detailed explanation). Ten to 100 µl aliquots of induced overnight cultures were diluted in 1 ml of phosphate-buffered saline and analyzed on a Becton-Dickenson FACSAria™ Fusion flow cytometer with excitation at 488 nm and FITC and Texas Red channel filters for detection of eGFP and mBeRFP fluorescence, respectively. Forward and side scattering gates were configured to select single-cell events and photomultiplier tube voltages were adjusted to 500–600 V to increase sensitivity for weak eGFP emission and to maximize the dynamic range of detection. Apparent TE was calculated from measured fluorescent protein emissions using the formula:

$$\mathrm{TE}=\left(1-{\left(\frac{\left\langle \left\langle \mathrm{mB}\right\rangle \right\rangle 0}{\left\langle \left\langle \mathrm{eG}\right\rangle \right\rangle 0}\right)\left(\frac{\left\langle \left\langle \mathrm{mB}\right\rangle \right\rangle \mathrm{Term}}{\left\langle \left\langle \mathrm{eG}\right\rangle \right\rangle \mathrm{Term}}\right)}^{-1}\right)\times 100\%$$

Where, ‘mB’ and ‘eG’ are the mBeRFP and eGFP fluorescence intensity, respectively. Clones containing pBeRG plasmids with terminator parts (‘Term’) were normalized to clones containing pBeRG plasmids that lack a terminator part (‘0’) to account for cell-to-cell plasmid copy number variation. Flow cytometry experiments were performed in duplicate with TE mean and standard deviation calculated from all cell events from each experiment. Plasmids from 50 selected clones from flow cytometry experiments were subjected to Sanger sequencing (Supplementary Table S2).

Fluorescence-activated cell sorting (FACS) experiments were performed as per flow cytometry, however, using entire *E. coli* terminator library samples. *E. coli* terminator library clones were sorted into four bins with gates corresponding to apparent TE values from 0% to 50% (weak terminators), 50–87% (intermediate-weak), 87–95% (intermediate-strong) and >95% (strong; Supplementary Figure S3A). Sorted cell fractions were used to inoculate 5 ml of fresh LB medium, grown to mid-log phase at 37°C with shaking and plated on LB_Amp100_ plates. Fractioned clone libraries were then cultivated, aliquoted and stored as described above.

### 2.4 Terminator RNAseq

Total RNA was isolated from terminator library *E. coli* cultures as described by Bernstein *et al.* (30) with modifications. Terminator library FACS-sorted library clones and control terminator control cultures were cultivated in LB_Amp100_ media overnight, sub-cultured into fresh LB_Amp100_ media, grown until mid-log phase (OD_600_ = 0.4) with shaking at 37°C and induced with 1 mM IPTG. Temperature-dependent termination experiments were performed as described above except library clones were incubated at 14°C, 21°C, 30°C or 37°C for 15 min prior to induction with continued incubation at the specified temperature for 1 h (Supplementary Figure S3B). For both FACS-sorted and temperature-dependent experiments, 5 ml aliquots of induced cultures were added to tubes containing 1 ml of ice-cold 95% ethanol containing and 5% saturated phenol (pH 6.8; to inhibit RNA degradation), rapidly mixed by vortexing, placed on ice and subsequently flash frozen in liquid nitrogen and stored at −80°C until required (less than 1 week). Total RNA was extracted via the hot phenolic extraction method followed by ethanol precipitation and elution in 50 µl of RNase-free water (MilliQ^®^). Contaminating DNA was removed by DNase I digestion in 20 µl reactions containing 10 µg of total RNA, 2 U DNase I (Thermo Scientific) and 1× DNase I reaction buffer and incubating at 37°C for 1 h followed by phenol extraction and ethanol precipitation and elution in 20 µl of RNase-free water (MilliQ^®^). RNA sample quality was assayed on 1% agarose gels and quantitated using a BioDrop™ spectrophotometer.

RNA samples were prepared for Illumina^®^ MiSeq Next Generation Sequencing using the NEBNext^®^ Multiplex Small RNA Library Prep Set for Illumina (New England Biolabs) with modifications (Supplementary Figure S3C). Because the 3ʹ end of reporter read-through transcripts would lie several hundred nucleotides downstream of the terminator cloning site, a new proximal 3ʹ end was generated for read-through transcripts by annealing an antisense DNA oligo that binds approximately 30 nt downstream from the terminator site and digesting with RNase H. Digestions were carried out in 20 µl reactions containing 10 µg of total RNA, 100 pmol antisense oligo (oTerm-Seq-R), 1× RNase H reaction buffer and 5 U RNase H (NEB) and incubated for 1 h at 37°C followed by phenol extraction and ethanol precipitation as described above. RNase H-treated samples were then prepared using the NEBNext^®^ Multiplex Small RNA Library Prep Set for Illumina (New England Biolabs) and barcoded as per the manufacturer’s instructions, except the 5ʹ linker ligation step was omitted. Library first-strand cDNAs were amplified using the P7 reverse primer and a custom gene-specific forward primer that binds ∼50 bp upstream of the terminator cloning site and bares the P5 oligo sequences at its 5ʹ end (oTerm-Seq-F5-P5). Barcoded terminator libraries were pooled and sequenced on an Illumina MiSeq Sequencer (GeneWiz) which yielded approximately 40 million, 2× 150 bp paired-end reads with 73% of reads ≥ Q30. Illumina adapters were removed using Cutadapt (31) with a threshold of 90% identity to adapter sequences. Only reads that passed quality control (≥Q30) and bared a complete T_hp_ sequence were considered for analysis (Supplementary Tables S3 and S4).

Unique terminator sequence identifiers were assigned with the designation ‘T’ and numbered in descending order of their sequence read abundance. Terminator counts were normalized for each sample replicate (in transcripts per million, TPM) and custom Python™ scripts (see Section 2.6) were used to tabulate transcript sequencing read lengths which served to determine whether transcripts were terminated or read-through. Transcripts were deemed as ‘terminated’ if their length corresponded to +1 to +8 nt downstream of the last nucleotide of the T_hp_ while transcripts with length greater than this (beyond the poly(U) tract) were counted as ‘read-through.’ The top 2000 terminators with the greatest number of total reads were then assessed for TE by counting the number of terminated transcripts divided by total reads for each terminator variant. Only terminator variants that had 200 or more reads in either FACS binning or differential temperature experiments are displayed (see Supplementary Tables S3 and S4 for all terminator sequences). T_hp_ minimal free-energy predications and predicted secondary structures were calculated for each terminator variant using the RNAfold program from the Vienna RNA Package (32) with folding parameters set to 37°C and -p -d2 –noLP modifiers.

Terminators identified in FACS-sorted terminator RNAseq (Term-Seq) libraries were normalized to TPM values, based on the total number of reads in each barcoded library. Terminators were examined for enrichment in FACS bins as per the bin with the greatest mean TPM values (most abundant bin), as well as by use of the two-tailed Student’s *t*-tests, which compared TPM counts from each FACS bin from triplicate biological replicates (*df* = 1; Supplementary Table S3). If *t*-test *P*-values were <0.05 for any one bin relative to all other bins, the terminator was considered significantly enriched in that bin. For differential temperature experiments, TE values for terminators were calculated for each temperature treatment from triplicate experiments. Individual terminator TEs were then compared for each temperature treatment using two-tailed Student’s *t*-tests for significant differences in terminator read-through efficiency (*P* < 0.05, *df =* 1).

### 2.6 Data availability

All custom scripts used for Term-Seq have been made available at GitHub (https://github.com/andyhudson42/TermSeq.git). Raw sequence data for Term-Seq were deposited and available from the NCBI Sequence Read Archive (SRA) under BioProject accession: PRJNA503821. All other relevant data are available upon request from the authors.

### 2.7 Materials availability

All DNA constructs will be provided upon request following the completion of a Materials Transfer Agreement and any other documentation that may be required.

## 3. Results

### 3.1 Rapid synthesis of intrinsic terminator libraries

Terminator libraries have been valuable tools for examining RNA/DNA sequence–function relationships and for establishing design principles for synthetic terminators with desirable TEs (5, 12). However, prior terminator library construction has required assembly of individually synthesized terminator parts, making the ability to probe the functional sequence space of even relatively small combinatorial libraries (∼100 sequences) both expensive and time-consuming. We, therefore, sought to develop methods to create libraries consisting of thousands of unique intrinsic terminator variants while maintaining control over features such as the length and composition of the T_hp_, poly(U) and poly(A) tracts and adjacent sequences (e.g. for cloning or part assembly purposes). Although we attempted several strategies for terminator library synthesis (many other strategies are likely possible), we found a strategy that uses two semi-randomized DNA oligonucleotides that provides a great compromise in terms of economy (could be performed rapidly and inexpensively, using readily available lab materials) and flexibility over terminator library design. In this method, two 5′ and 3′ half oligos are designed using a computer to contain desired features (see Supplementary Note S1 for design considerations) and generated by conventional DNA oligo synthesis. Oligos are then assembled into terminator libraries in four steps (see Section 2.1).

**Figure 1.**
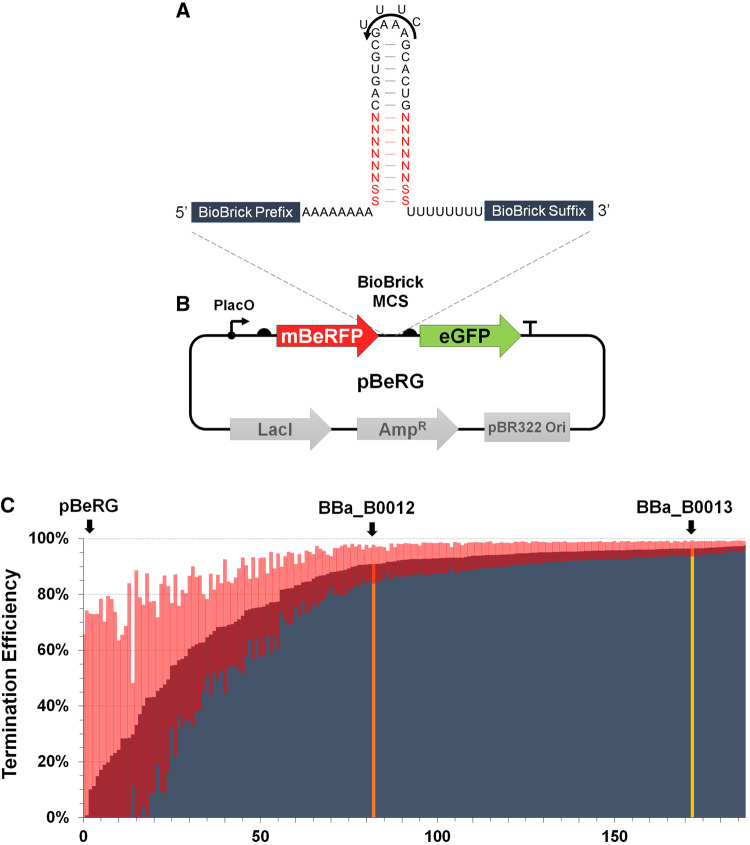
Intrinsic terminator library synthesis and measurement by flow cytometry. (**A**) Terminator library design used in this study. The corresponding RNA sequence for the terminator library is indicated with boxes representing BioBrick^®^ RFC 10 prefix and suffix sequences. The randomized portion of the T_hp_ is in red text, where ‘S’ indicates a ‘C’ or ‘G’ nucleotide and ‘N’ is any nucleotide. The forward T_hp_ loop sequence is shown with the reverse sequence shown above. (**B**). Schematic of the bi-cistronic pBeRG terminator testing device is shown using SBOL visual format and indicates the terminator cloning site (BioBrick multicloning site, MCS). See Supplementary Figure S1 for pBeRG vector sequence. (**C**) Flow cytometry measurements for 180 *E. coli* NEB5α terminator library clones were performed in duplicate and TE values were calculated (see Methods section). Standard deviations are shown for clones as red lines and the pBeRG (without terminator, first bar on left), BBa_B0012 terminator and BBa_B0013 terminator clones are indicated as blue, orange and gold bars. See Supplementary Table S2 for terminator sequences.

Because strong intrinsic terminators (>90% TE) with diverse sequences are particularly desirable for insulating synthetic cistrons, we applied our terminator library methodology to create very strong intrinsic terminators: a 14 bp, perfectly paired hairpin with a basal 8 bp randomized portion that is closed by two strong (G-C or C-G) pairs, a GAAA tetraloop (or UUUC in reverse), a downstream 8 nt poly(U) tract and an upstream 8 nt poly(A) tract (12) (Figure 1A). We placed BioBrick^®^ RFC 10 standard prefix and suffix sequences in the upstream and downstream constant regions to allow the library parts to be easily assembled into genetic circuits via BioBrick^®^ assembly (http://parts.igem.org/Assembly: Standard_assembly). Our library design has a theoretical maximum diversity of 16 384 sequences and once designed, terminator library assembly could be performed in a single 8-h day.

### 3.2 Synthetic terminator library characterization via flow cytometry

We cloned our terminator library nondirectionally into a custom bi-cistronic terminator testing device (pBeRG) at a BioBrick^®^ RFC 10 multicloning site (Figure 1B) and examined TEs of terminators from our library (*n* = ∼10 000 clones) using a fluorescence-based interference strategy and flow cytometry. Flow cytometry revealed a bimodal distribution for all terminator library clones and two distinct populations with median fluorescence interference TEs (designated TE_IF_) of 13.6% and 96.5%, relative to no terminator (Figure 1C and Supplementary Figure S2). For comparison, we benchmarked terminators from our library against two previously characterized intrinsic terminators: BBa_B0012 (medium-efficiency terminator) and BBa_B0013 (high-efficiency terminator) from the Registry of Standardized Biological Parts (http://parts.igem.org). In pBeRG, flow cytometry experiments predicted 90.9% and 96.5% TE_IF_ for the BBa_B0012 and BBa_B0013 terminators, respectively (Figure 1C and Supplementary Table S2). TEs for 180 library clones were then measured individually using flow cytometry. More than half of the measured library terminators displayed apparent TE_IF_ of 90% or greater and many terminators showed even higher efficiency than the strong BBa_B0013 terminator (Figure 1C). Fifty clones were Sanger sequenced, which identified only unique terminator variants and confirmed successful terminator library cloning with sufficient diversity to warrant further examination using higher throughput methods (Supplementary Table S2).

### 3.3 High-throughput measurement of terminators using qTerm-Seq

Flow cytometry-based methods have been routinely applied to examine TEs of intrinsic terminators *in vivo* (5, 12); however, they are less amenable to high-throughput examination of terminators (e.g. hundreds of terminators) due to the need to individually clone and measure terminator parts. Transcriptome-based methods are highly scalable and an attractive alternative to flow cytometry for higher throughput studies examining transcription termination, due to the ability to examine thousands of transcripts in a single experiment. Term-Seq is a recently described RNAseq method that maps RNA transcript 3′ ends to identify transcription termination regulators in *E. coli* (22) and other bacteria (21). However, while Term-Seq identifies putative termination sites, it has not been applied to quantitate TE. Therefore, we developed a quantitative Term-Seq method and bioinformatic strategy (qTerm-Seq) that can simultaneously identify terminator sequences and evaluate transcription TE of terminators from our synthetic library (see Section 2 and Supplementary Figure S3).

To determine whether TEs from flow cytometry-based (TE_IF_) were comparable to those obtained by qTerm-Seq (TE_TS_), we used FACS to separate terminator library clones into four fractions prior to qTerm-Seq with FACS gates corresponding to: 0% to 50% TE (Weak terminators), 50–87% TE (Intermediate-Weak), 87–95% TE (Intermediate-Strong) and >95% TE (Strong).

qTerm-Seq identified over 10 000 unique library sequences; however, some of these are expected to represent cloning or sequencing artifacts (e.g. only single-read representation). We examined the distribution of 3′ ends for all qTerm-seq reads relative to the pBeRG terminator reporter construct to identify putative transcription termination sites (Supplementary Figure S4A). Transcript 3ʹ end positions showed similar distributions for all four FACS bins, with exception of weak terminators, which suggests that terminators separated into the remaining bins had similar transcriptional and degradation profiles *in vivo* (Supplementary Figure S4A). A large proportion of the qTerm-Seq reads were 3ʹ truncated within the 5′ and 3′ arms of the T_hp_ portion of terminator library sequences (Supplementary Figure S4A). Although this collection may indeed represent *bona fide* transcription termination sites or stable reporter mRNA degradation products, a complete T_hp_ sequence could not be determined for these reads and they were removed from further analysis. A peak +1 to +3 nt downstream of the T_hp_ was also evident and due to their close proximity to the expected termination site for intrinsic terminators (+6 to +8 nt downstream of T_hp_) (23) were deemed to have arisen from transcription termination events (Supplementary Figure S4A). qTerm-Seq reads whose 3ʹ ends extended beyond +8 nt downstream of the T_hp_ were classified as read-through events in which RNA polymerase did not terminate at the candidate terminator.

**Figure 2.**
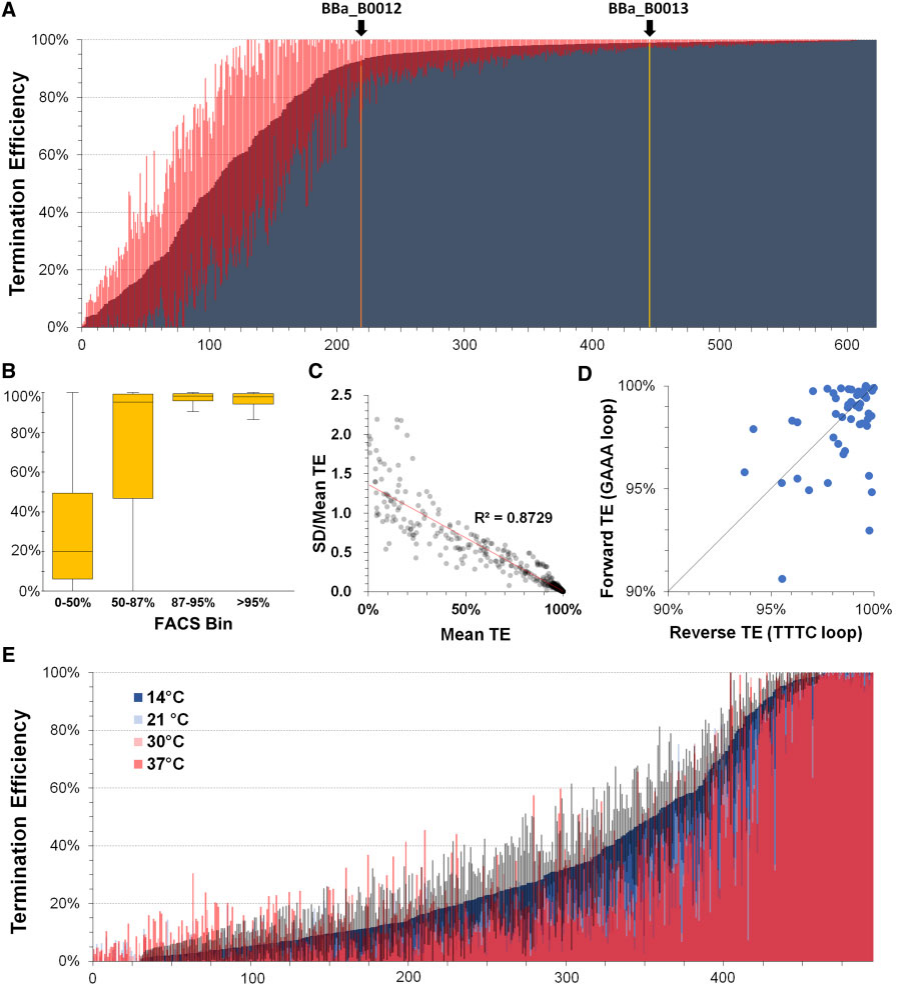
High-throughput measurement of library terminators via qTerm-Seq. **(A**) TEs for 622 unique library terminators (>200 reads) from FACS binning experiments were determined using qTerm-Seq and are compared with those determined for the BBa_B0012 (orange bar) and BBa_B0013 (gold bar) test terminators. Red lines indicate TE standard deviations from three biological replicates. See Supplementary Table S3 for terminator sequences and other data and Supplementary Figure S5 for additional details. (**B**) Boxplot comparing qTerm-Seq-calculated TE values for terminators in each FACS bin. Box boundaries represent the upper and lower quartiles (median indicated as a horizontal line within) and vertical lines indicate extreme upper and lower TE values. (**C**) Relationship of mean TE with standard deviation normalized to mean TE. (**D**) Comparison of terminator strength for terminators measured using qTerm-Seq in forward and reverse orientations. (**E**) qTerm-Seq measurement of 495 library terminators at various expression temperatures. Terminator variants are ordered from low to high mean TE based on the 14°C sample data with corresponding mean TE measured for the 21°C, 30°C and 37°C overlaid. Gray bars indicate standard deviations from three biological replicates for the 14°C sample data. See Supplementary Table S4 for terminator sequences and other data and Supplementary Figure S6 for additional details.

After removing truncated reads with an incomplete T_hp_ and terminator sequences with fewer than 200 reads, 622 unique full-length terminator variants remained for analysis (Supplementary Table S3). The overall distribution of terminator library TE_TS_ from the 622 FACS-enriched terminators mimics the distribution obtained from individual flow cytometry experiments (cf. Figures 1C and 2A). Terminator library TE_TS_ predicted by qTerm-Seq also corresponded closely with enrichment in the four FACS bins (Figure 2B), indicating that qTerm-Seq and flow cytometry measurements generate comparable apparent TEs.

TE_TS_ measurement for the BBa_B0012 and BBa_B0013 terminators was 92.8% (SD = 1.9%) and 99.1% (SD = 0.21%), respectively and ∼2–3% higher than TE_IF_ values predicted by flow cytometry experiments for the two terminators. Unfortunately, none of the ∼50 sequences obtained from Sanger sequencing were present in the 622 terminators measured in qTerm-Seq and these could not be directly compared using the two methods.

From the collection of 622 FACS-enriched terminators, 423 terminators had a TE_TS_ greater than 90% and 180 terminators were greater than 99% (Figure 2A and Supplementary Figure S5 and Table S3). The strongest 26 terminators had TE_TS_ greater than 99.9%, corresponding to more than a 1000-fold difference in read-through, relative to the lowest measured terminators from the library. As expected for our library design, most strong terminators featured a perfectly base-paired T_hp_. Weak terminators (< 50% TE_TS_) were found to be mostly terminator assembly or cloning artifacts that could not form a strong hairpin structure, and this is consistent with the importance of T_hp_ base pairing integrity for efficient intrinsic termination (24). Consistent with prior studies (12, 33), we observed that nucleotide substitutions that disrupt base pairing within the middle of the T_hp_ reduced TE_TS_ to a lesser extent than the two closing T_hp_ base pairs and nucleotide insertions between the T_hp_ and the first uridine of the poly(U) tract drastically reduced TE_TS_. As observed in other studies (5, 12), TE_TS_ variance was inversely proportional to terminator strength, with the strongest terminators consistently having lower variance in TE_TS_ between experimental replicates (Figure 2C).

Fifty-two terminators were measured in both forward and reverse orientations and differed only in their hairpin loop sequence (either a ‘GAAA’ or ‘TTTC’; Supplementary Table S3). Neither loop sequence was consistently linked with increased or decreased TE_TS,_ nor differences in forward and reverse TE_TS_ values were typically smaller than standard error between experimental replicates (Figure 2D and Supplementary Table S3). However, stronger terminators generally showed less variation between their forward and reverse orientations than weaker terminators and several terminators maintained greater than 99% TE_TS_ in both directions (Figure 2D and Supplementary Table S3).

### 3.4 Expression temperature does not strongly affect TE_TS_ of library terminators

Terminator hairpin folding dynamics during transcription can be affected by expression temperature and plausibly this may confer changes in terminator efficiency. Therefore, we also expressed our terminator library at 14°C, 21°C, 30°C and 37°C in *E. coli* prior to qTerm-Seq to examine possible temperature-dependent termination effects in our library (Supplementary Figure S3B). After removing low-abundance terminators (<200 reads), we identified 495 terminator variants, which did not overlap significantly with those from the FACS-enriched terminator pool (Supplementary Table S4). Although measured terminator TE_TS_ spanned two orders of magnitude (0.62–99.96%), most of the terminators did not display significant differences in TE at expression temperatures from 14°C to 37°C and very strong terminators showed the least temperature-dependent variance in TE_TS_ (Figure 2E and Supplementary Figure S6). Patterns of qTerm-Seq 3ʹ end positions did not show obvious deviations for different expression temperatures, except at an upstream site within the BioBrick^®^ prefix sequence which occurred more frequently at 14°C and 21°C (Supplementary Figure S4B). Several terminators were found that had significantly different TE_TS_ from 14°C to 37°C (Figure 2E and Supplementary Table S4) and most of these exhibited a higher TE_TS_ at lower temperature with the largest difference being a 78% decrease at 14°C as compared with 37°C (T528).

### 3.5 Terminator features correlating to TE

**Figure 3.**
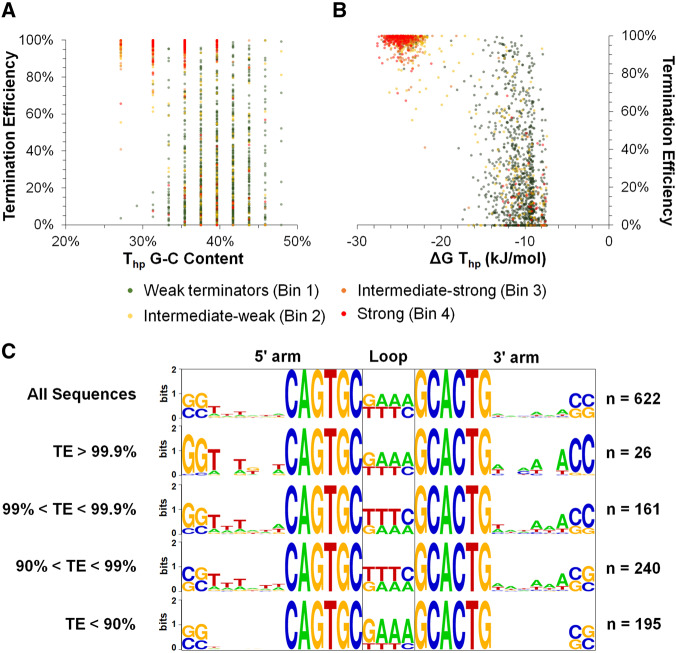
Sequence and structural features affecting intrinsic terminator strength. (**A, B**) Terminator hairpin G-C content and predicted free-energy of folding are examined with respect to TE. Colored dots indicate the corresponding FACS bin that terminators were enriched (see Supplementary Figure S3A for gating strategy). Terminator hairpin free-energies were calculated using RNAFold from the Vienna RNA Package (32). (**C**) Sequence logo representations (WebLogo (34)) of terminator hairpins sequence conservation for terminators with dependent on TE. Nucleotide letter heights indicate their frequency at each position of the terminator hairpin.

Using the collection of library terminators measured by qTerm-Seq, we looked for terminator features that correlated to TE_TS_. T_hp_ predicted free-energies somewhat correlated with TE_TS_ and there was an apparent division in T_hp_ free-energy with terminators with a ΔG of less than −20 kJ/mol having TEs greater than 90% and terminators with greater ΔG typically having lower TE (Figure 3A and B and Supplementary Tables S3 and S4). Terminator hairpin G/C content did not show any obvious relationship to TE_TS_, although surprisingly, the strongest terminators in the library generally had less G/C-rich hairpins (Figure 3A). It is possible that PCR amplification of the terminator library and our qTerm-Seq library preparation methods favored more A/T-rich terminators and that even stronger (more G/C rich) terminators may exist in our library but escaped detection using qTerm-Seq.

Because our terminator library only varies the proximal 8 bp of the T_hp_, we were able to specifically examine the effect of basal hairpin sequence composition on TE_TS_ in greater depth (Figure 1A). Terminators were divided into four pools with 10-fold increment TE_TS_ cut offs (<90%, >90%, >99%, >99.9%) to examine possible conserved sequence features of each pool. The strongest measured terminators (TE >99%) showed a high frequency of 5ʹ-GG…CC-3ʹ as the closing base pairs of the T_hp_ (128 out of 187 terminators), while less strong terminators (< 99%) did not conserve this motif (115 out of 435 terminators; Figure 3C). Conservation of a 5ʹ-G…C-3ʹ closing base pair for very strong terminators was also noted by Cambray and colleagues (Arkin and Endy Laboratories, BIOFAB) (5) in a library of natural and synthetic intrinsic terminators.

## 4. Discussion

Synthetic biologists will require even greater numbers of characterized biological parts than are currently available and more accurate design parameters to create elaborate multigene devices and synthetic genomes. Here, we assist with this bottleneck by introducing novel methods for creating sequence-diverse libraries of bidirectional intrinsic terminators and developed quantitative Term-Seq to evaluate the performance of hundreds to thousands of unique intrinsic terminator parts in a single experiment. In our proof-of-concept, we employed qTerm-Seq to characterize a synthetic terminator library with strong terminator design features in *E. coli* at various growth temperatures. We find that most strong terminators from out synthetic library produce stable TEs from expression temperatures from 14°C to 37°C and our collection of characterized synthetic terminators adds hundreds of characterized BioBrick^®^-compatible transcription terminators that reduce read-through by up to 1000-fold. We did not explore the TE of our library terminators in other genetic and cellular contexts; however, we expect that our library design and qTerm-Seq methods can be utilized to identify library terminators with required functional properties *in vivo* for a wide variety of genetic contexts and cellular conditions.

Reuse of common biological parts in large genetic assemblies contributes to undesirable homologous recombination at repeat regions and limits design complexity. Consequently, large numbers of sequence-diverse terminators are needed to reduce the occurrence of gene rearrangements, insertions and deletions in synthetic genetic circuits (12). Due to flexibility of our terminator library method, it is now possible to create disparate terminator libraries that have low sequence similarity, enabling selection of terminators with desired properties while reducing the possibility of homologous recombination. The ability to manipulate the length and sequence of the T_hp_ stem and loop sequence as well as the other terminator features will facilitate more detailed studies that examine specific terminator DNA/RNA features that influence transcription termination. This is in particular interesting as it will allow the simple design of intrinsic terminator libraries for a variety of RNA polymerases, either from different host organism or in a purified and reconstituted system, creating the framework for optimal gene circuit design for uses *in vitro* or in specific host organisms. Our construction pipeline addresses, therefore, one of the founding challenges in synthetic biology, focusing on improving the reliability of design by using libraries of well-characterized parts and their development.

## Supplementary Material

ysz026_Supplementary_Data
