# Supplementary material for: Rapid generation of sequence-diverse terminator libraries and their parameterization using quantitative Term-Seq
Source: Synth Biol (Oxf). 2019 Oct 29;4(1):ysz026. doi: 10.1093/synbio/ysz026 (PMC7445774; doi:10.1093/synbio/ysz026)
Supplement: ysz026_Supplementary_Data [file ysz026_supplementary_data.zip › Terminator Library Manuscript - Supplementary Figures and Notes.docx]

**Rapid Generation of Sequence-diverse Terminator Libraries and their Parameterization using Quantitative TermSeq**

Andrew J. Hudson^1,2^, and Hans-Joachim Wieden*^1,3^

^1^Alberta RNA Research and Training Institute (ARRTI), University of Lethbridge, Lethbridge, Alberta, Canada

^2^ Department of Biological Sciences, University of Lethbridge, Lethbridge, Alberta, Canada

^3^ Department of Chemistry and Biochemistry, University of Lethbridge, Lethbridge, Alberta, Canada

* Corresponding Author

| **Supplementary Item** | **Description** | **Page** |
| --- | --- | --- |
| Supplementary Figure 1 | pBeRG Plasmid Map and Sequence | 2 |
| Supplementary Figure 2 | Representative Flow Cytometry Results for Terminators and Controls in pBeRG | 5 |
| Supplementary Figure 3 | Sample preparation workflow for qTermSeq | 6 |
| Supplementary Figure 4 | qTerm-Seq Transcript 3’ End Mapping | 7 |
| Supplementary Figure 5 | FACS-sorted qTerm-Seq Library Terminators | 8 |
| Supplementary Figure 6 | Differential Temperature qTerm-Seq Library Terminators | 11 |
| Supplementary Note 1 | Terminator Library Assembly and Design Considerations | 15 |
| Supplementary Note 2 | Rationale for Extended Cell Incubation After Induction and Before Flow Cytometry Analysis | 17 |

**Supplemental Figure 1 – pBeRG Plasmid Map and Sequence**


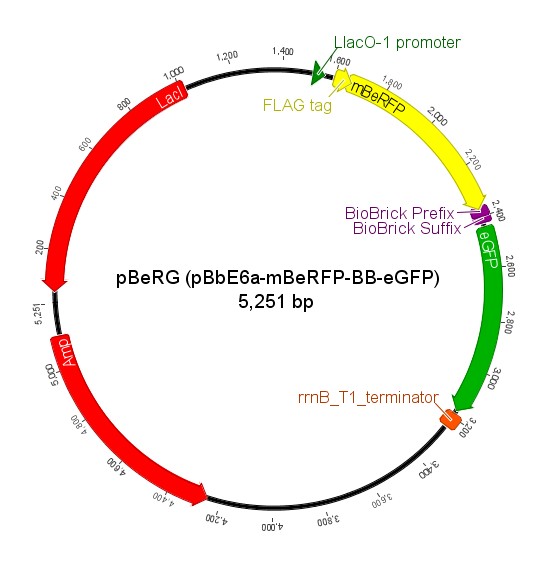


**pBeRG - pBR322 origin plasmid (15-20 copies)**

1 10 20 30 40 50

| | | | | |

GACGTCGGTGCCTAATGAGTGAGCTAACTTACATTAATTGCGTTGCGCTC

ACTGCCCGCTTTCCAGTCGGGAAACCTGTCGTGCCAGCTGCATTAATGAA

TCGGCCAACGCGCGGGGAGAGGCGGTTTGCGTATTGGGCGCCAGGGTGGT

TTTTCTTTTCACCAGTGAGACGGGCAACAGCTGATTGCCCTTCACCGCCT

GGCCCTGAGAGAGTTGCAGCAAGCGGTCCACGCTGGTTTGCCCCAGCAGG

CGAAAATCCTGTTTGATGGTGGTTAACGGCGGGATATAACATGAGCTGTC

TTCGGTATCGTCGTATCCCACTACCGAGATGTCCGCACCAACGCGCAGCC

CGGACTCGGTAATGGCGCGCATTGCGCCCAGCGCCATCTGATCGTTGGCA

ACCAGCATCGCAGTGGGAACGATGCCCTCATTCAGCATTTGCATGGTTTG

TTGAAAACCGGACATGGCACTCCAGTCGCCTTCCCGTTCCGCTATCGGCT

GAATTTGATTGCGAGTGAGATATTTATGCCAGCCAGCCAGACGCAGACGC

GCCGAGACAGAACTTAATGGGCCCGCTAACAGCGCGATTTGCTGGTGACC

CAATGCGACCAGATGCTCCACGCCCAGTCGCGTACCGTCTTCATGGGAGA

AAATAATACTGTTGATGGGTGTCTGGTCAGAGACATCAAGAAATAACGCC

GGAACATTAGTGCAGGCAGCTTCCACAGCAATGGCATCCTGGTCATCCAG

CGGATAGTTAATGATCAGCCCACTGACGCGTTGCGCGAGAAGATTGTGCA

CCGCCGCTTTACAGGCTTCGACGCCGCTTCGTTCTACCATCGACACCACC

ACGCTGGCACCCAGTTGATCGGCGCGAGATTTAATCGCCGCGACAATTTG

CGACGGCGCGTGCAGGGCCAGACTGGAGGTGGCAACGCCAATCAGCAACG

ACTGTTTGCCCGCCAGTTGTTGTGCCACGCGGTTGGGAATGTAATTCAGC

TCCGCCATCGCCGCTTCCACTTTTTCCCGCGTTTTCGCAGAAACGTGGCT

GGCCTGGTTCACCACGCGGGAAACGGTCTGATAAGAGACACCGGCATACT

CTGCGACATCGTATAACGTTACTGGTTTCACATTCACCACCCTGAATTGA

CTCTCTTCCGGGCGCTATCATGCCATACCGCGAAAGGTTTTGCGCCATTC

GATGGTGTCCGGGATCTCGACGCTCTCCCTTATGCGACTCCTGCATTAGG

AAGCAGCCCAGTAGTAGGTTGAGGCCGTTGAGCACCGCCGCCGCAAGGAA

TGGTGCATGCAAGGAGATGGCGCCCAACAGTCCCCCGGCCACGGGGCCTG

CCACCATACCCACGCCGAAACAAGCGCTCATGAGCCCGAAGTGGCGAGCC

CGATCTTCCCCATCGGTGATGTCGGCGATATAGGCGCCAGCAACCGCACC

TGTGGCGCCGGTGATGCCGGCCACGATGCGTCCGGCGTAGAGGATCGAGA

ATTGTGAGCGGATAACAATTGACATTGTGAGCGGATAACAAGATACTGAG

CACATCAGCAGGACGCACTGACCGAATTCGCGTAAAATCAATAAGGAGAC

AACAAGATGGACTACAAAGACGATGACGACAAGAGCAGCGGCCTGGTGCC

GCGCGGCAGCATGGCTAGCGGCGAAGAGCTGATTAAGGAGAACATGCACA

TGAAGCTGTACATGGAGGGCACCGTGAACAACCACCACTTCAAGTGCACA

TCCGAGGGCGAAGGCAAGCCCTACGAGGGCACCCAGACCATGAGAATCAA

GGTGGTCGAGGGCGGCCCTCTCCCCTTCGCCTTCGACATCCTGGCTACCA

GCTTCATGTACGGCAGTAAGACCTTCATCAACCACACCCAGGGCATCCCC

GACTTCTTCAAGCAGTCCTTCCCTGAGGGCTTCACATGGGAGAGATCCAC

CACATACGAAGACGGGGGCGTGCTGACCGCTACCCAGGACACCAGCCTCC

AGGACGGCTGCCTCATCTACAACGTCAAGATCAGAGGGGTGAACTTCCCA

TCCAACGGCCCTGTGATGCAGAAGAAAACACTCGGCTGGGAGGCCAGCAC

CGAGATGCTGTACCCCGCTGACGGCGGCCTGGAAGGCAGAGACTACATGG

CCCTGAAGCTCGTGGGCGGGGGCCACCTGATCTGCAACGCTAAGACCACA

TACAGATCCAAGAAACCCGCTAAGAACCTCAAGATGCCCGGCGTCTACTA

TGTGGACAGAAGACTGGAAAGAATCAAGGAGGCCGACAAAGAGACCAGCG

TCGAGCAGCACGAGGTGGCTGTGGCCAGATACTGCGACCTCCCTAGCAAA

CTGGGGCACAAATAAGACAGAATTCGCGGCCGCTTCTAGAGGGATCCTAC

TAGTAGCGGCCGCTGCAGTGCTAAAATCAATAAGGAGACAACCATATGGT

GAGCAAGGGCGAGGAGCTGTTCACCGGGGTGGTGCCCATCCTGGTCGAGC

TGGACGGCGACGTAAACGGCCACAAGTTCAGCGTGTCCGGCGAGGGCGAG

GGCGATGCCACCTACGGCAAGCTGACCCTGAAGTTCATCTGCACCACCGG

CAAGCTGCCCGTGCCCTGGCCCACCCTCGTGACCACCCTGACCTACGGCG

TGCAGTGCTTCAGCCGCTACCCCGACCACATGAAGCAGCACGACTTCTTC

AAGTCCGCCATGCCCGAAGGCTACGTCCAGGAGCGCACCATCTTCTTCAA

GGACGACGGCAACTACAAGACCCGCGCCGAGGTGAAGTTCGAGGGCGACA

CCCTGGTGAACCGCATCGAGCTGAAGGGCATCGACTTCAAGGAGGACGGC

AACATCCTGGGGCACAAGCTGGAGTACAACTTCAACAGCCACAACGTCTA

TATCATGGCCGACAAGCAGAAGAACGGCATCAAGGTGAACTTCAAGATCC

GCCACAACATCGAGGACGGCAGCGTGCAGCTCGCCGACCACTACCAGCAG

AACACCCCCATCGGCGACGGCCCCGTGCTGCTGCCCGACAACCACTACCT

GAGCACCCAGTCCGCCCTGAGCAAAGACCCCAACGAGAAGCGCGATCACA

TGGTCCTGCTGGAGTTCGTGACCGCCGCCGGGATCACTCTCGGCATGGAC

GAGCTGTACAAGTAACTCGAGTAAGGATCTCCAGGCATCAAATAAAACGA

AAGGCTCAGTCGAAAGACTGGGCCTTTCGTTTTATCTGTTGTTTGTCGGT

GAACGCTCTCTACTAGAGTCACACTGGCTCACCTTCGGGTGGGCCTTTCT

GCGTTTATACCTAGGGCGTTCGGCTGCGGCGAGCGGTATCAGCTCACTCA

AAGGCGGTAATACGGTTATCCACAGAATCAGGGGATAACGCAGGAAAGAA

CATGTGAGCAAAAGGCCAGCAAAAGGCCAGGAACCGTAAAAAGGCCGCGT

TGCTGGCGTTTTTCCATAGGCTCCGCCCCCCTGACGAGCATCACAAAAAT

CGACGCTCAAGTCAGAGGTGGCGAAACCCGACAGGACTATAAAGATACCA

GGCGTTTCCCCCTGGAAGCTCCCTCGTGCGCTCTCCTGTTCCGACCCTGC

CGCTTACCGGATACCTGTCCGCCTTTCTCCCTTCGGGAAGCGTGGCGCTT

TCTCATAGCTCACGCTGTAGGTATCTCAGTTCGGTGTAGGTCGTTCGCTC

CAAGCTGGGCTGTGTGCACGAACCCCCCGTTCAGCCCGACCGCTGCGCCT

TATCCGGTAACTATCGTCTTGAGTCCAACCCGGTAAGACACGACTTATCG

CCACTGGCAGCAGCCACTGGTAACAGGATTAGCAGAGCGAGGTATGTAGG

CGGTGCTACAGAGTTCTTGAAGTGGTGGCCTAACTACGGCTACACTAGAA

GGACAGTATTTGGTATCTGCGCTCTGCTGAAGCCAGTTACCTTCGGAAAA

AGAGTTGGTAGCTCTTGATCCGGCAAACAAACCACCGCTGGTAGCGGTGG

TTTTTTTGTTTGCAAGCAGCAGATTACGCGCAGAAAAAAAGGATCTCAAG

AAGATCCTTTGATCTTTTCTACGGGGTCTGACGCTCAGTGGAACGAAAAC

TCACGTTAAGGGATTTTGGTCATGACTAGTGCTTGGATTCTCACCAATAA

AAAACGCCCGGCGGCAACCGAGCGTTCTGAACAAATCCAGATGGAGTTCT

GAGGTCATTACTGGATCTATCAACAGGAGTCCAAGCGAGCTCGTAAACTT

GGTCTGACAGTTACCAATGCTTAATCAGTGAGGCACCTATCTCAGCGATC

TGTCTATTTCGTTCATCCATAGTTGCCTGACTCCCCGTCGTGTAGATAAC

TACGATACGGGAGGGCTTACCATCTGGCCCCAGTGCTGCAATGATACCGC

GAGACCCACGCTCACCGGCTCCAGATTTATCAGCAATAAACCAGCCAGCC

GGAAGGGCCGAGCGCAGAAGTGGTCCTGCAACTTTATCCGCCTCCATCCA

GTCTATTAATTGTTGCCGGGAAGCTAGAGTAAGTAGTTCGCCAGTTAATA

GTTTGCGCAACGTTGTTGCCATTGCTACAGGCATCGTGGTGTCACGCTCG

TCGTTTGGTATGGCTTCATTCAGCTCCGGTTCCCAACGATCAAGGCGAGT

TACATGATCCCCCATGTTGTGCAAAAAAGCGGTTAGCTCCTTCGGTCCTC

CGATCGTTGTCAGAAGTAAGTTGGCCGCAGTGTTATCACTCATGGTTATG

GCAGCACTGCATAATTCTCTTACTGTCATGCCATCCGTAAGATGCTTTTC

TGTGACTGGTGAGTACTCAACCAAGTCATTCTGAGAATAGTGTATGCGGC

GACCGAGTTGCTCTTGCCCGGCGTCAATACGGGATAATACCGCGCCACAT

AGCAGAACTTTAAAAGTGCTCATCATTGGAAAACGTTCTTCGGGGCGAAA

ACTCTCAAGGATCTTACCGCTGTTGAGATCCAGTTCGATGTAACCCACTC

GTGCACCCAACTGATCTTCAGCATCTTTTACTTTCACCAGCGTTTCTGGG

TGAGCAAAAACAGGAAGGCAAAATGCCGCAAAAAAGGGAATAAGGGCGAC

ACGGAAATGTTGAATACTCATACTCTTCCTTTTTCAATATTATTGAAGCA

TTTATCAGGGTTATTGTCTCATGAGCGGATACATATTTGAATGTATTTAG

AAAAATAAACAAATAGGGGTTCCGCGCACATTTCCCCGAAAAGTGCCACC

T

**
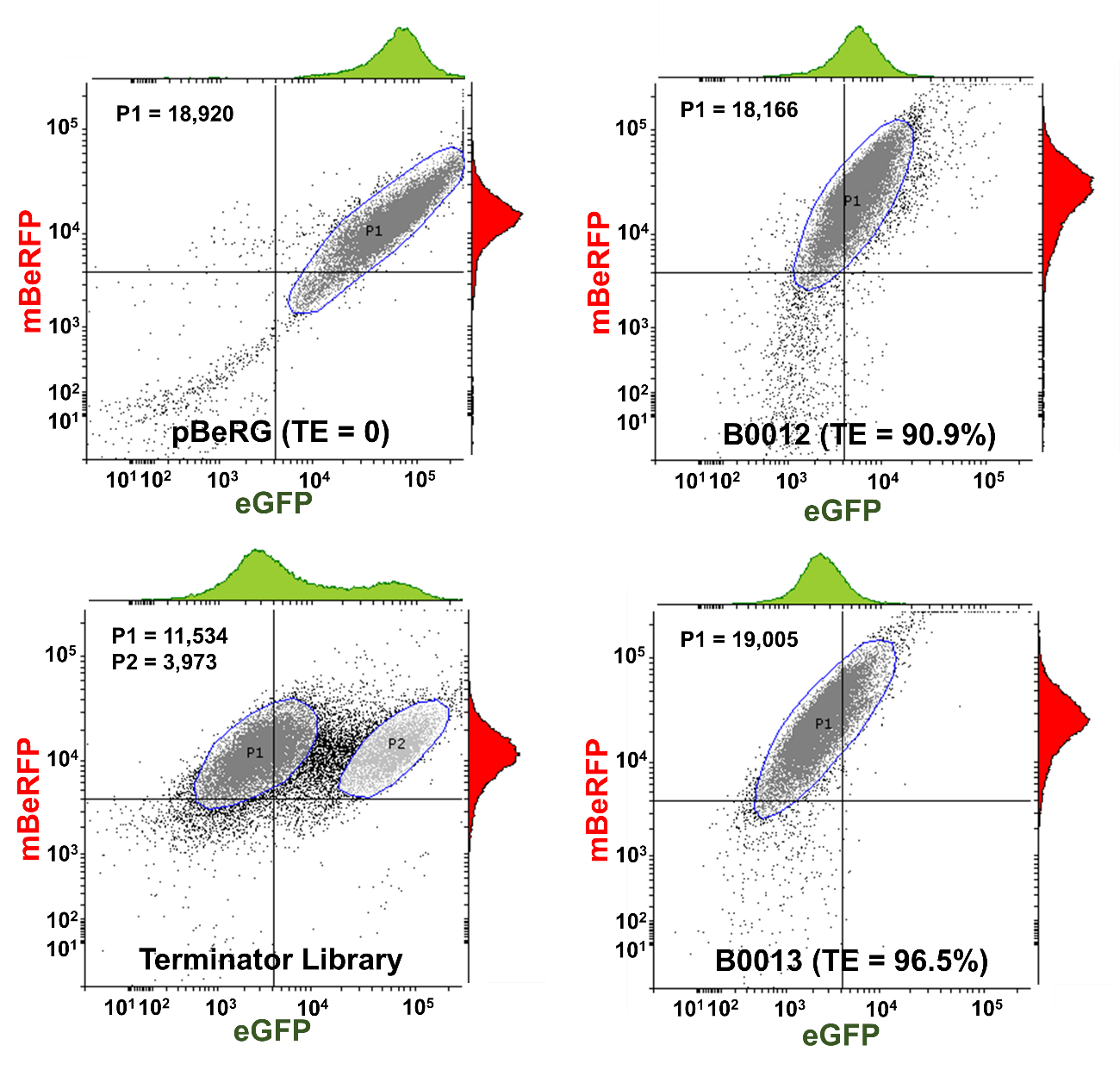
**

**B0013**

**pBeRG**

**Supplemental Figure 2. Representative Flow Cytometry Results for Terminators and Controls in pBeRG**

Dot plots displaying the fluorescent intensities of eGFP (X-axis, green trace) and mBeRFP (Y-axis, red trace) are compared for *E. coli* NEB5α clones expressing pBeRG with terminator library and with clones expressing pBeRG without terminator or the previously characterized BBa_B0012 or BBa_B0013 terminators from the Registry of Standardized Biological Parts (<http://parts.igem.org/Terminators/Catalog>). For the terminator library panel, the population distributions for pBeRG alone and for pBeRG plus the BBa_B0013 terminator are overlaid as green and red ellipses, respectively.

**
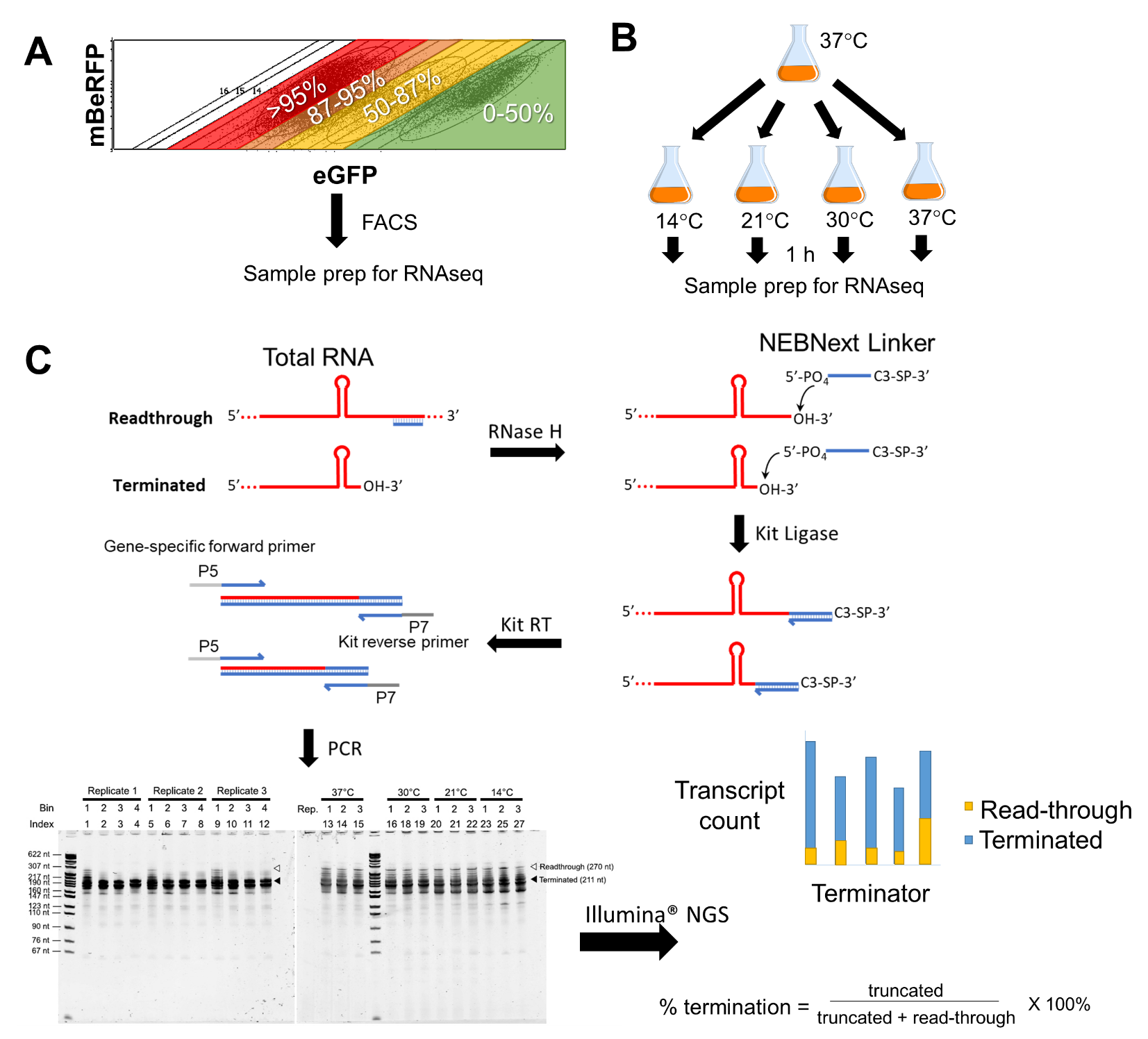
**

**Supplemental Figure 3. Sample preparation workflow for qTerm-Seq**

**A.** FACS gating strategy used prior to total RNA preparation of terminator expressing clones. Clones were separated into four bins (n = 10^6^ events per bin) that correspond to 0-50% TE (green gate), 50-87% TE (yellow), 87-95% TE (orange) and >95% TE (red). **B.** Temperature dependence of terminators expressed at 14°C, 21°C 30°C or 37°C. **C.** Total RNA from *E. coli* clones was then prepared for TermSeq using the NEBNext® Small RNA Library Prep Set for Illumina^®^ with modifications (see Online Methods). An antisense DNA oligo was annealed to read-through transcripts followed by digestion with RNase H to generate a proximal 3′ end for 3′ linker ligation. RNA was then reverse transcribed using an oligo that binds the 3′ end linker and then PCR-amplified using custom P5 forward and P7 reverse primers (see Supplementary Table 1 for primer sequences).

**A. qTerm-Seq FACS Binning Experiments**

**Discarded transcripts (incomplete terminator hairpin sequence)**

**Terminator Sequence**

**eGFP CDS start codon**

**BioBrick Suffix**

**BioBrick Prefix**

**Readthrough transcripts**

**Terminated**

**transcripts**

**B. qTerm-Seq Differential Temperature Experiments**

**Terminator Sequence**

**eGFP CDS start codon**

**BioBrick Suffix**

**BioBrick Prefix**

**Readthrough transcripts**

**Terminated**

**transcripts**

**Discarded transcripts (incomplete terminator hairpin sequence)**

**Supplemental Figure 4. qTerm-Seq Transcript 3’ End Mapping**

Frequencies for transcript 3ʹ end positions within the pBeRG-terminator library construct for FACS binning experiments (**A**) and differential temperature experiments (**B**) are shown, indicating distributions for FACS binned clone sequences and RNA samples from *E. coli* cells expressed at 14°C to 37°C. Nucleotide sequences for the region containing the terminator multiple cloning site of pBeRG is displayed with the degenerate terminator library sequence shown and the 5ʹ end of the eGFP coding sequences is indicated downstream. Transcript reads whose 3ʹ ends lie between 0 to +8 nt downstream of the T_hp_ were deemed as terminated while transcript reads with 3ʹ end +9 nt and beyond were deemed as readthrough transcripts. Transcript reads that did not contain a complete T_hp_ sequence were indeterminate and were excluded from qTerm-Seq analysis.

**Supplemental Figure 5 – FACS-sorted qTerm-Seq Library Terminators**


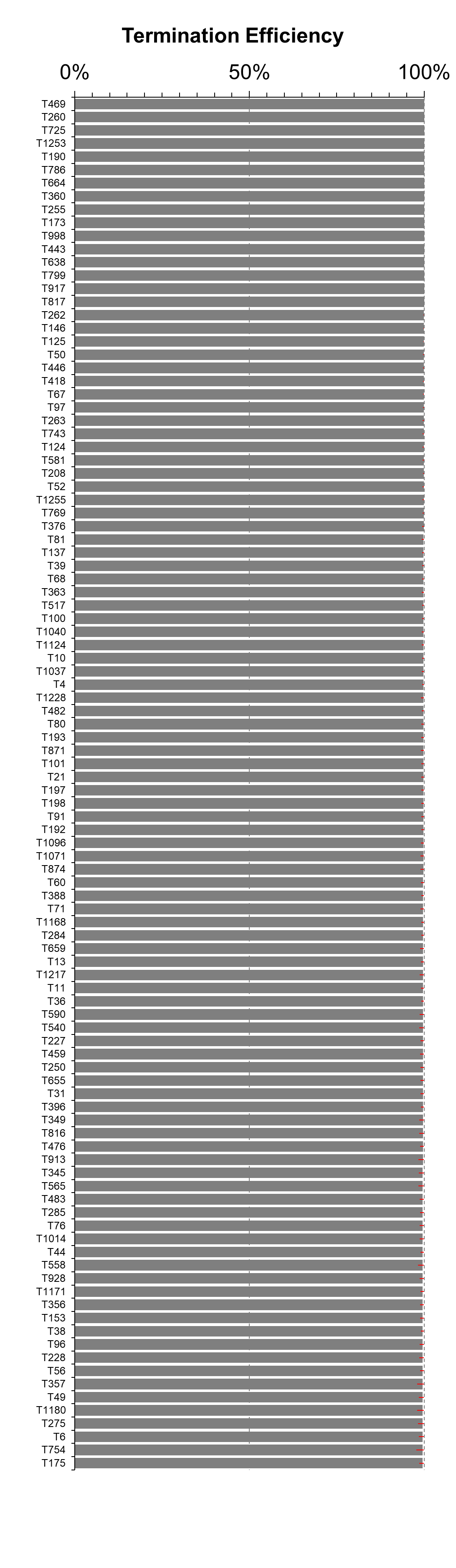

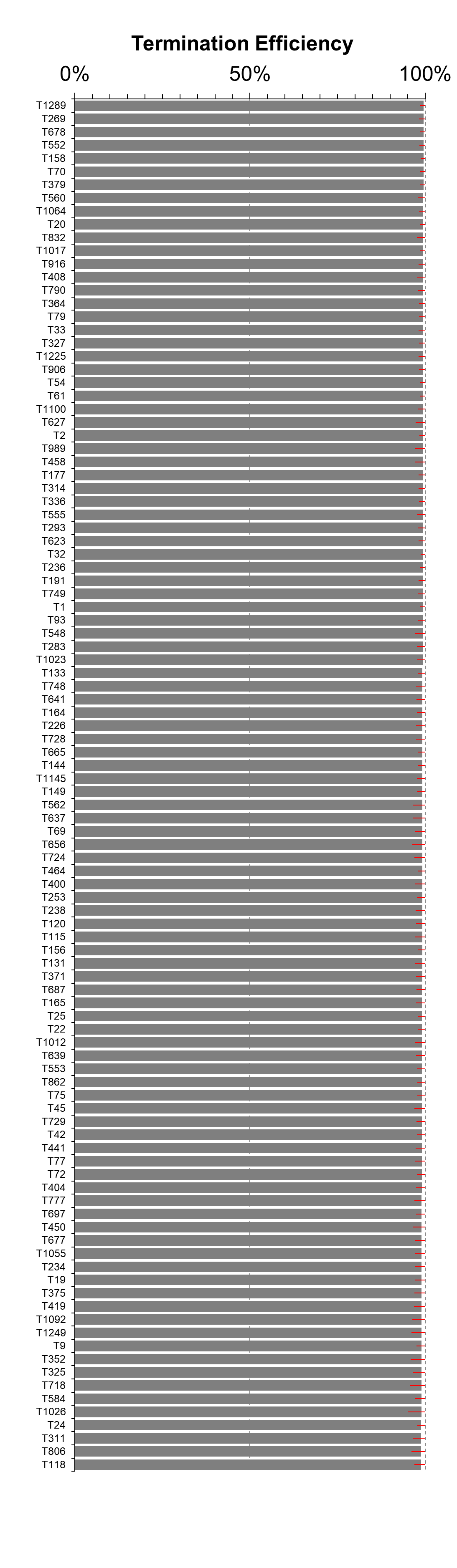

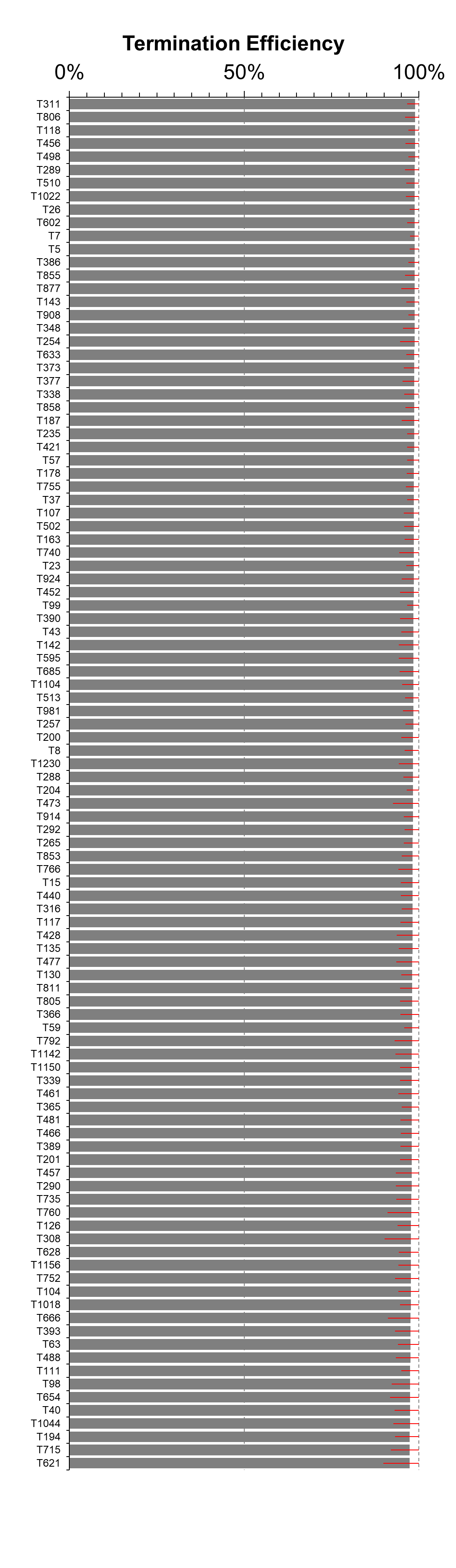


**Supplemental Figure 5 – FACS-sorted qTerm-Seq Library Terminators (Continued)**


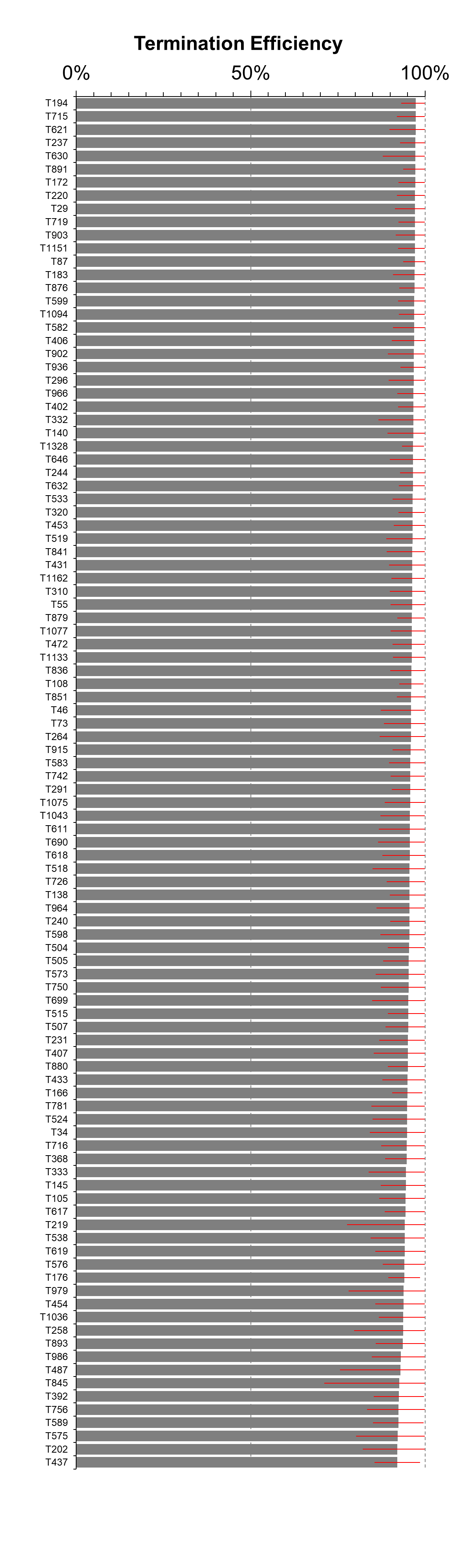

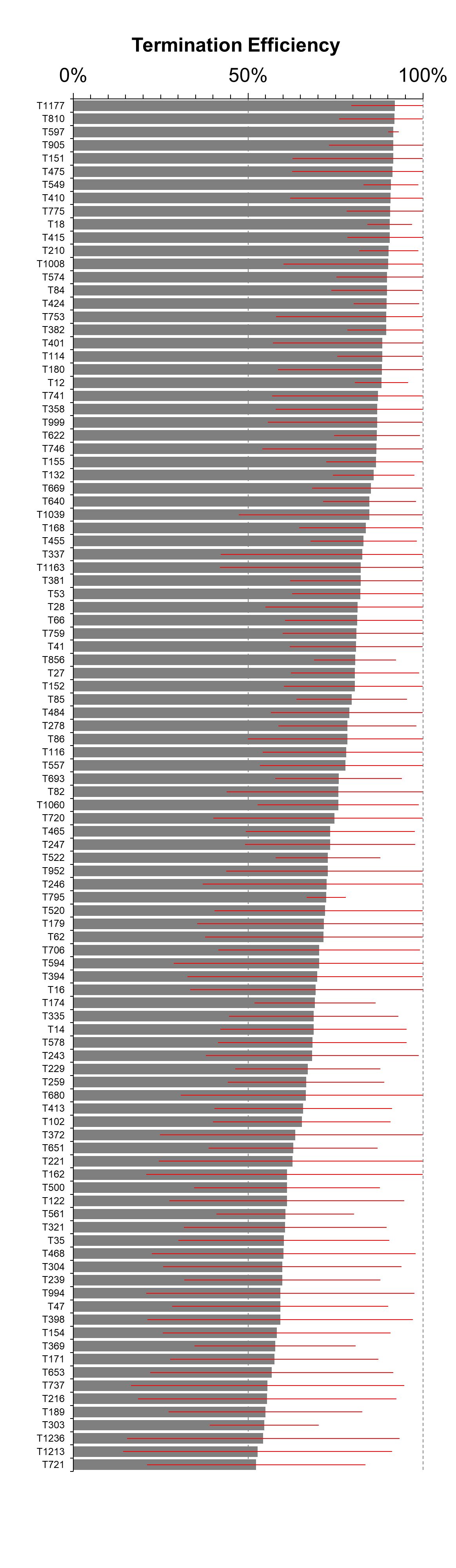

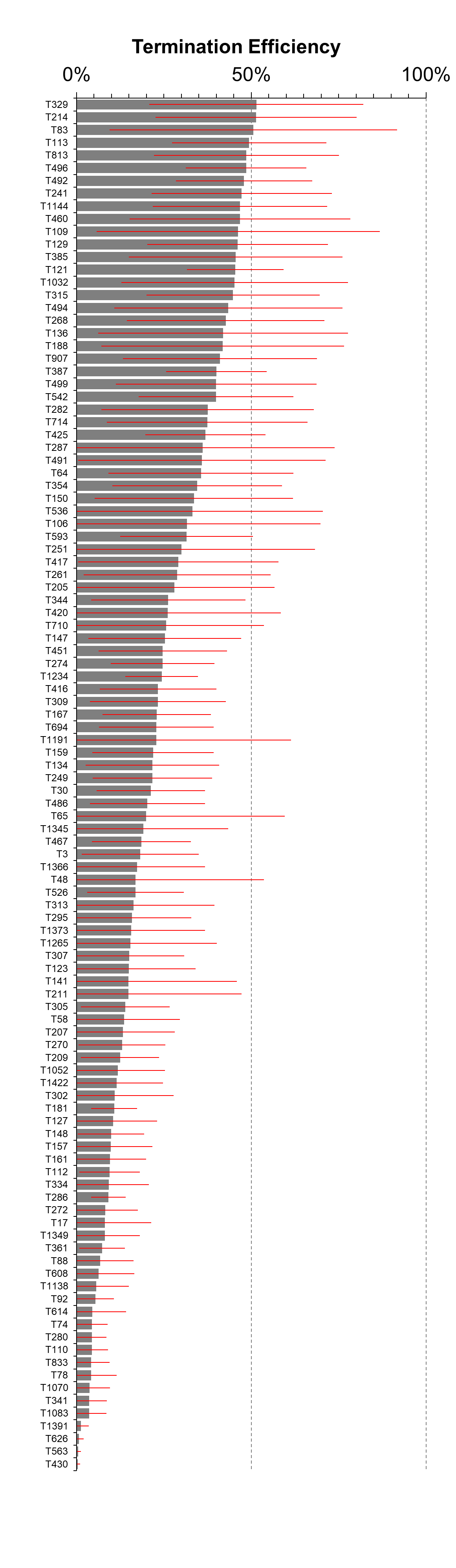


**Supplemental Figure 5. FACS-sorted qTerm-Seq Library Terminators.**

Termination efficiencies for terminators identified from qTermSeq from FACS-sorted libraries are shown with red bars indicating standard deviations from three experimental replicates. See Supplemental Table 3 for terminator sequences and additional data.

**Supplemental Figure 6 – Differential Temperature qTerm-Seq Library Terminators**


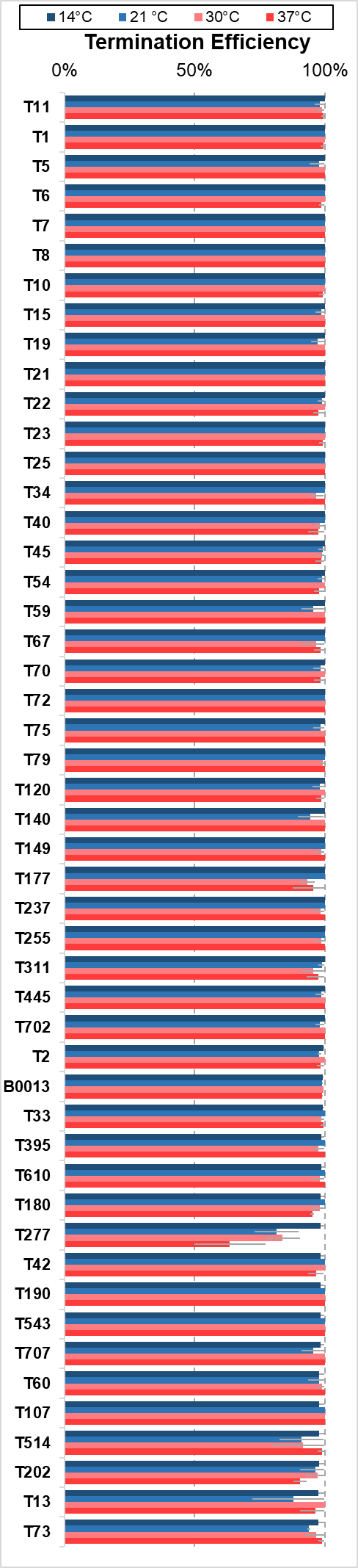

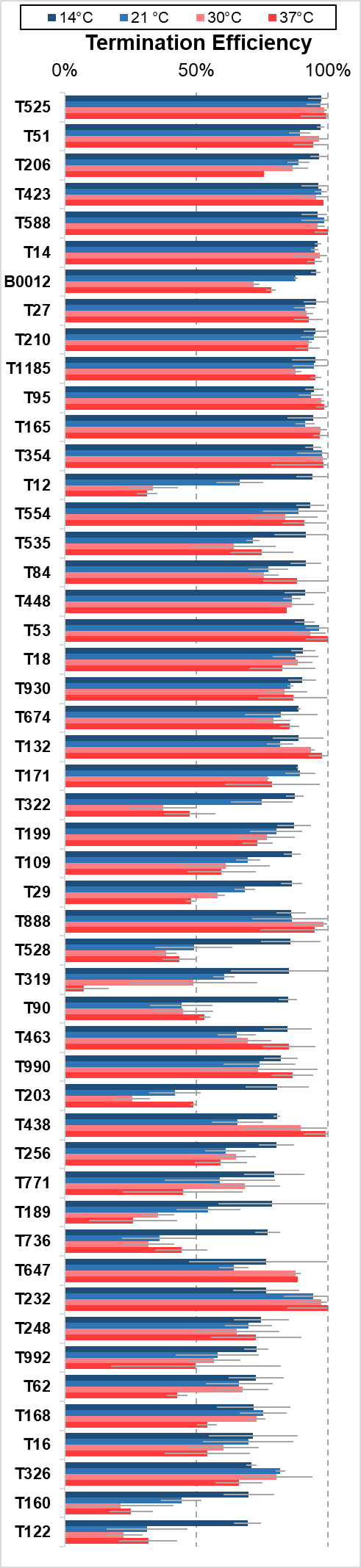

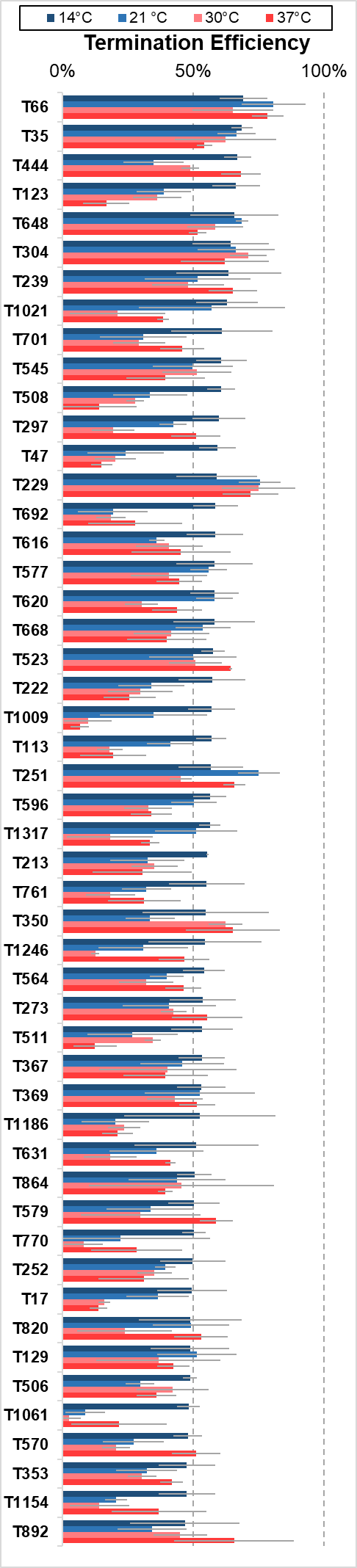

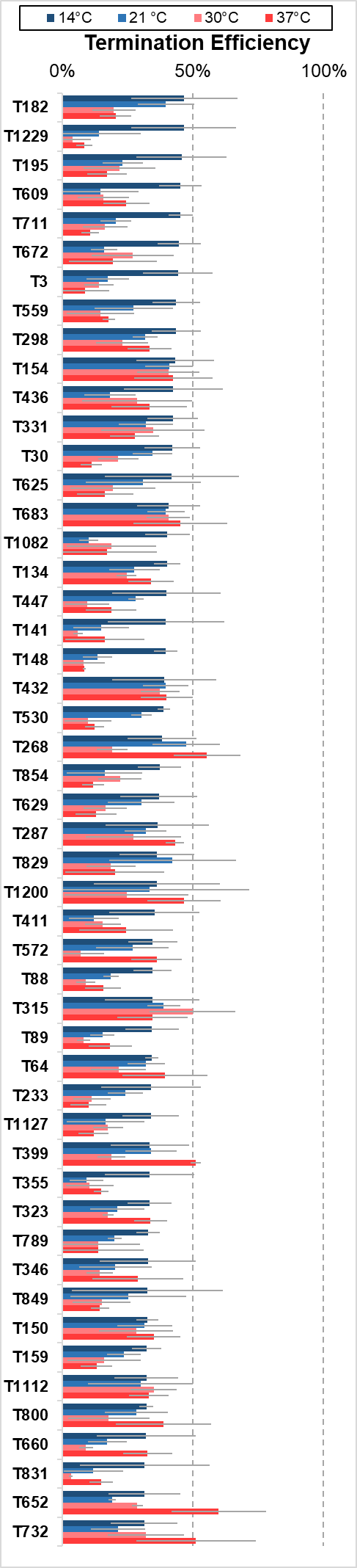


**Supplemental Figure 5 – Differential Temperature qTerm-Seq Library Terminators (Continued)**


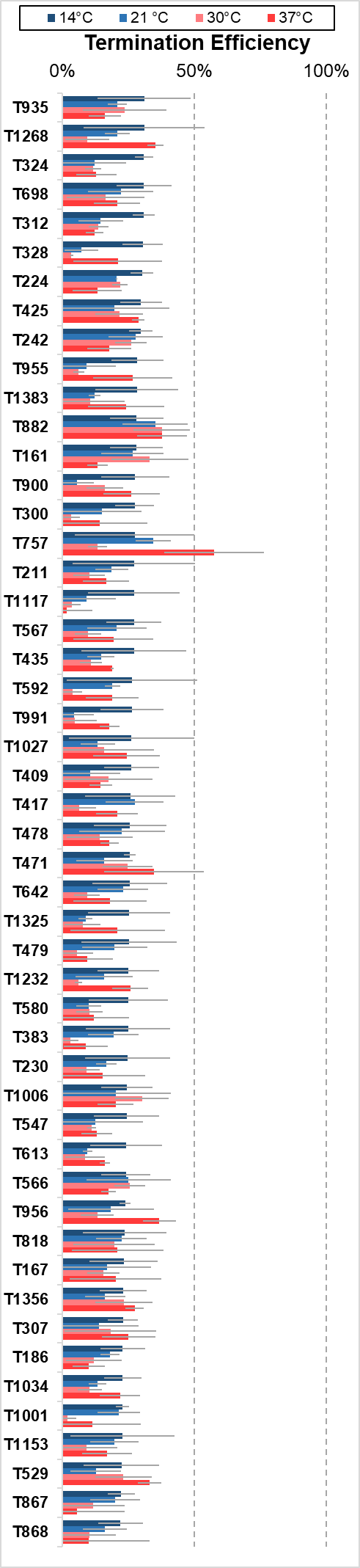

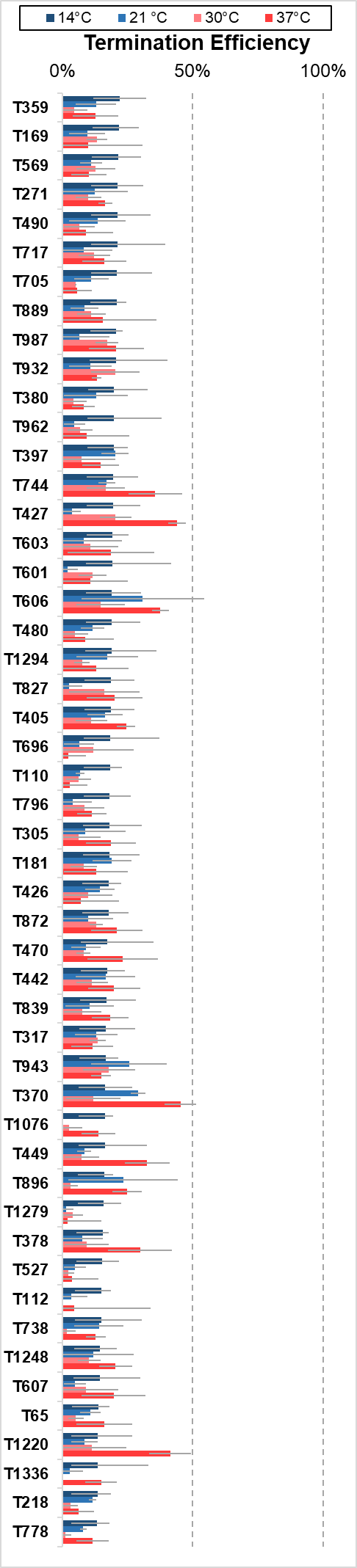

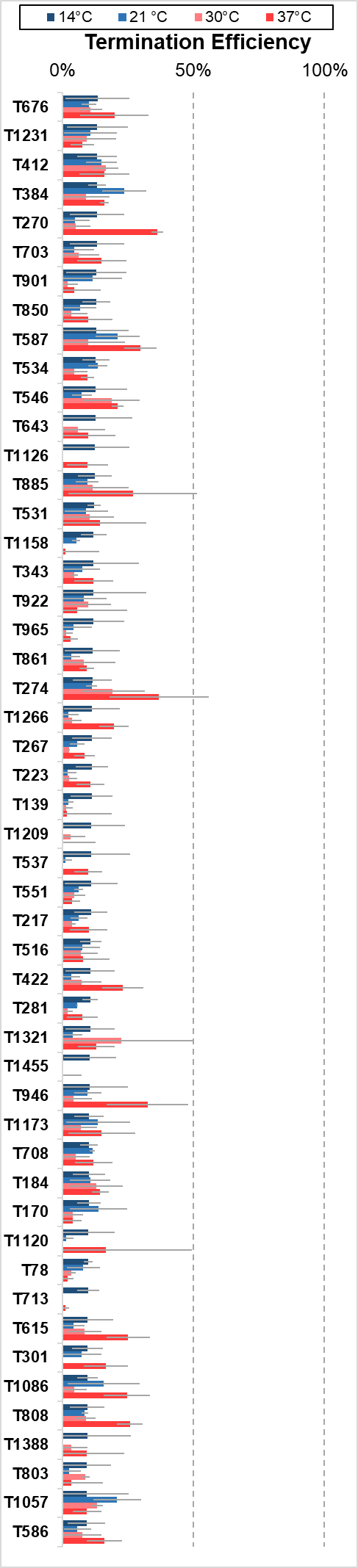

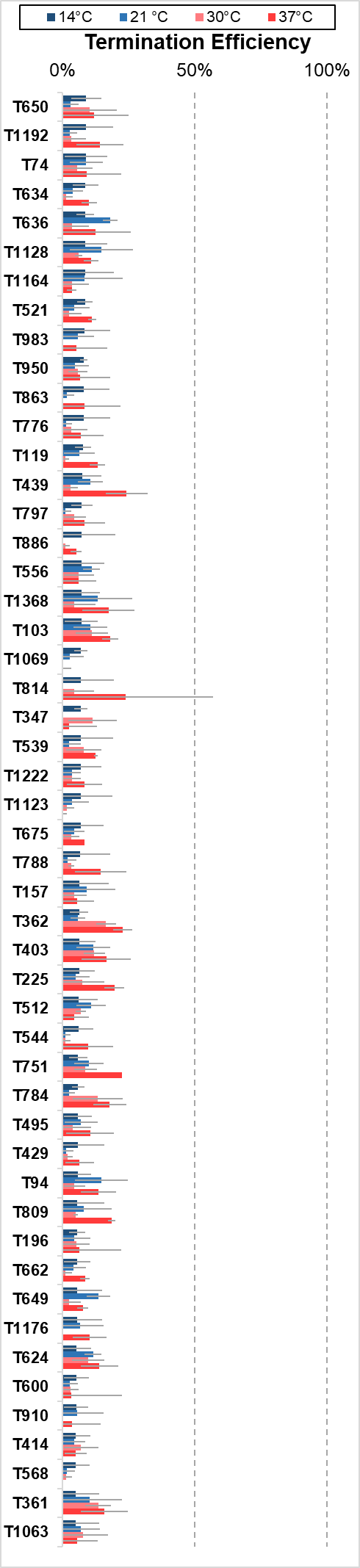


**Supplemental Figure 5 – Differential Temperature qTerm-Seq Library Terminators (Continued)**


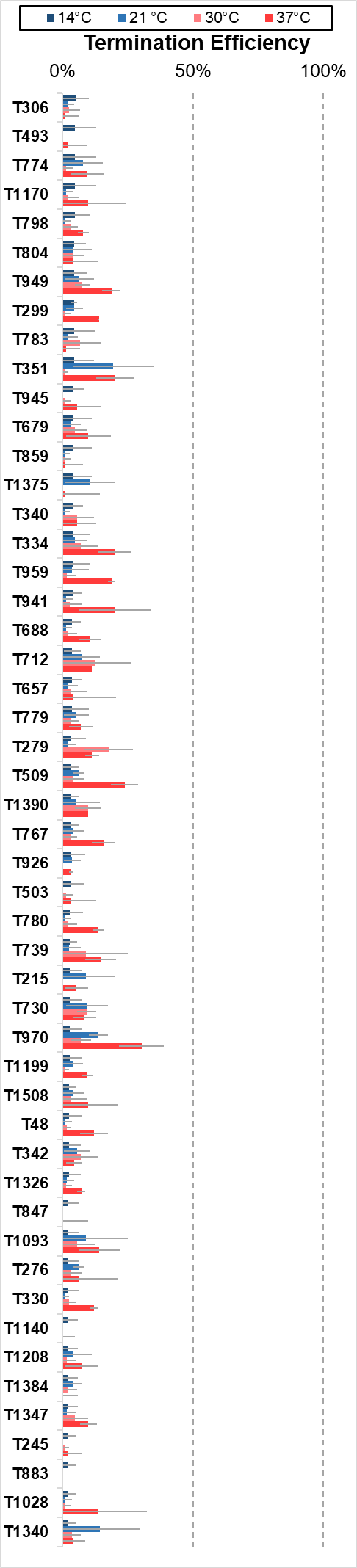

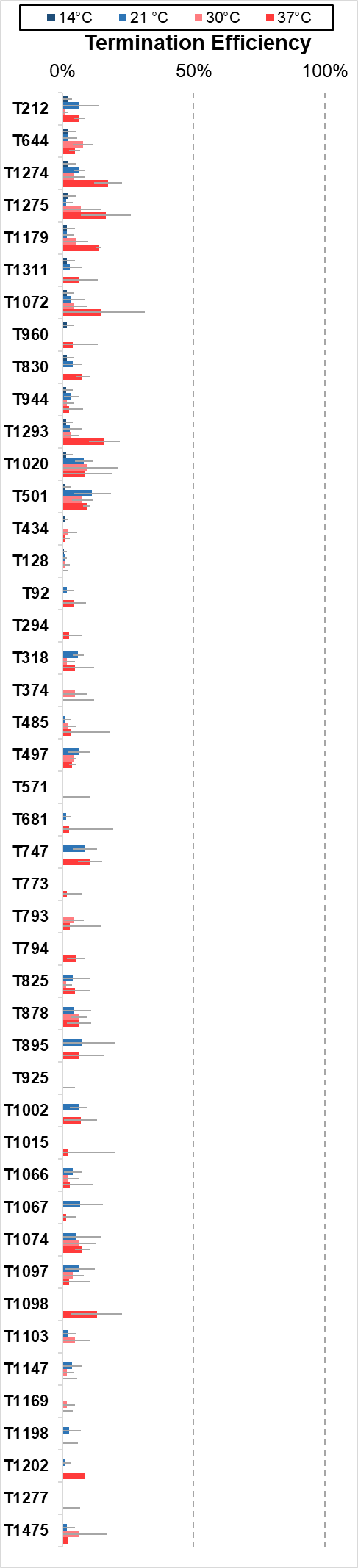


**Supplemental Figure 5. Differential Temperature qTerm-Seq Library Terminators.**

Termination efficiencies for terminators from differential temperature qTerm-Seq libraries are shown for expression temperatures of 14°C (Dark Blue), 21°C (Light Blue), 30°C (Salmon) and 37°C (Red). Standard deviations are indicated for each terminator temperature as gray bars from three experimental replicates. See Supplemental Table 4 for terminator sequences and additional data.

**Supplementary Note 1 – Terminator Library Assembly and Design Considerations**


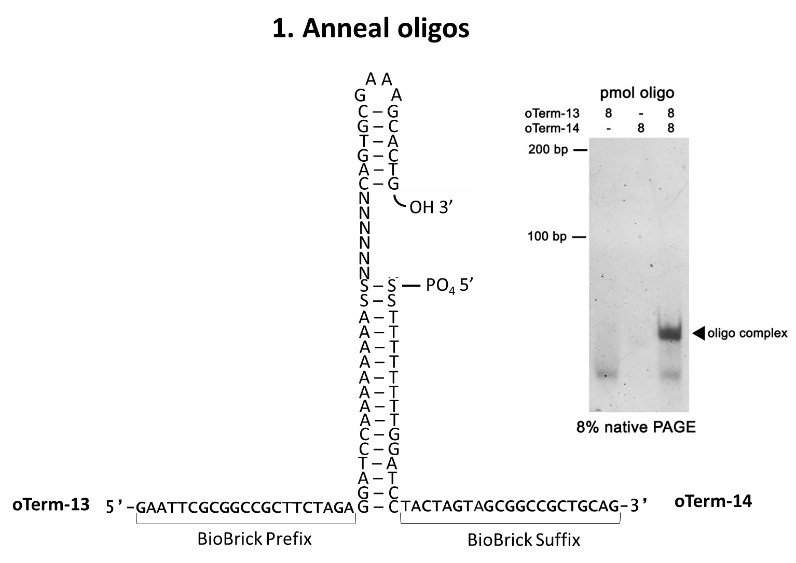

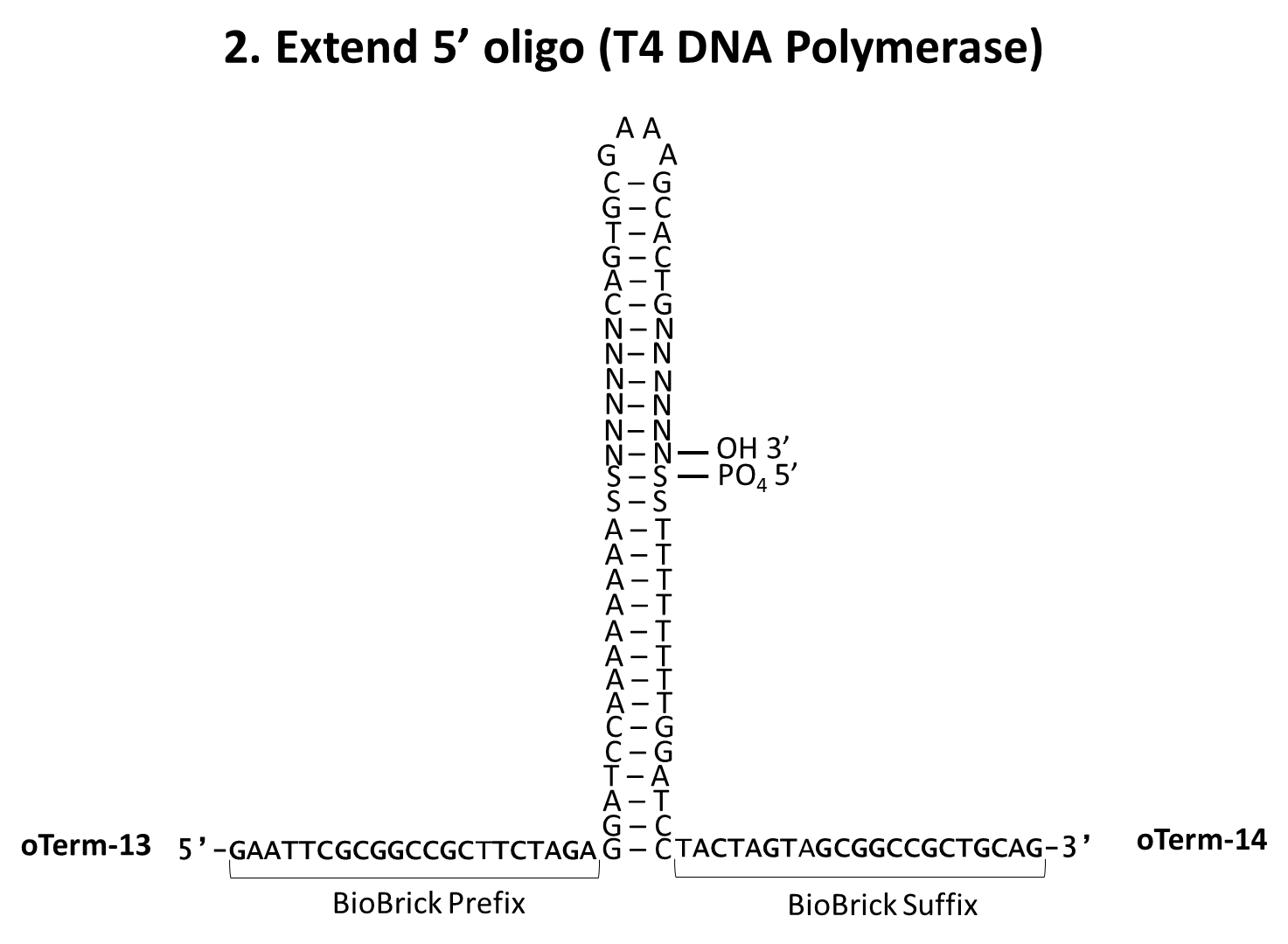


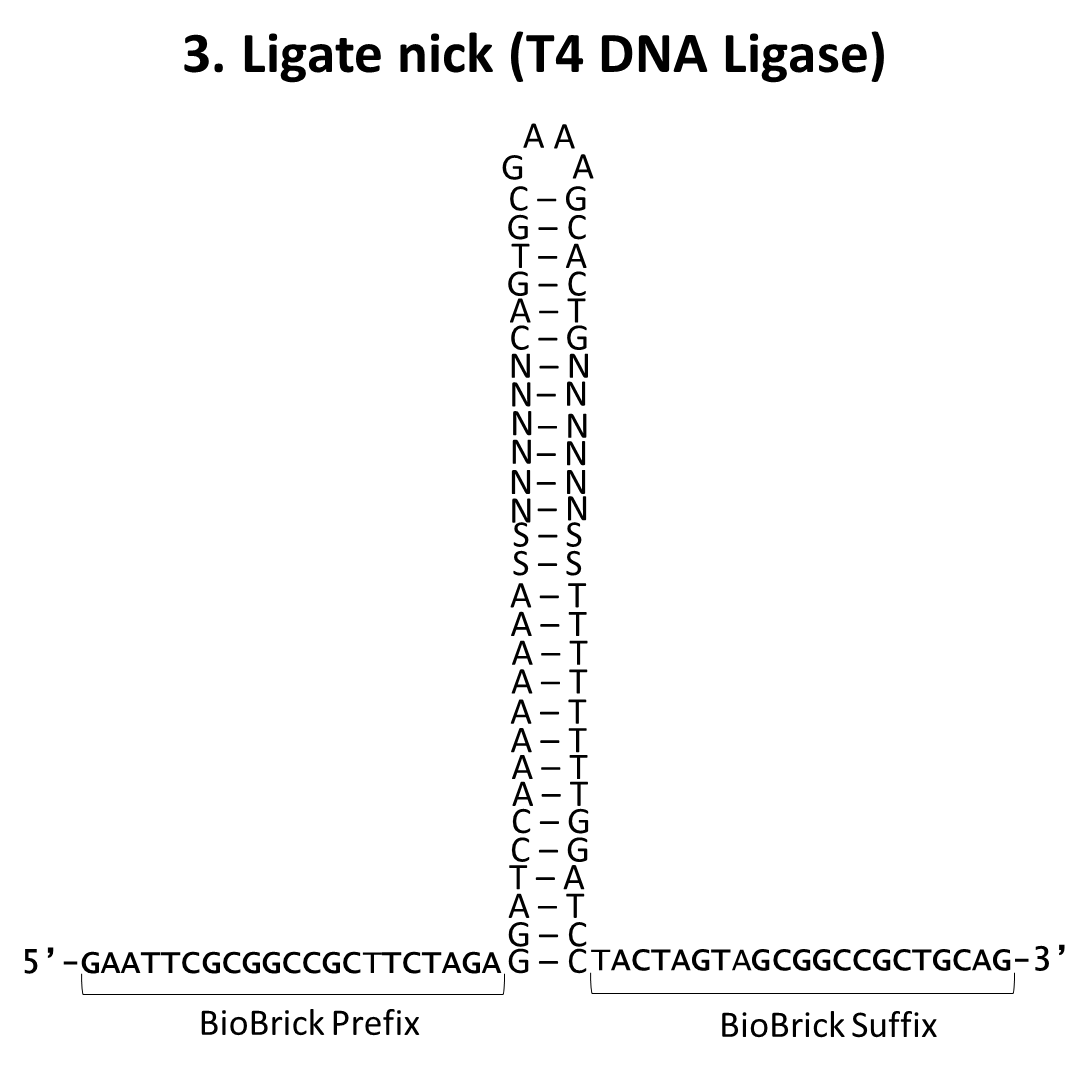

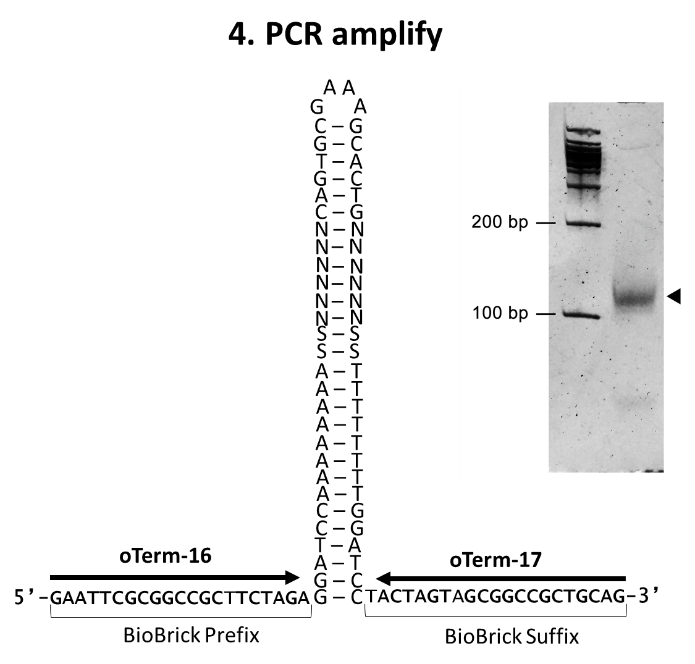


Terminator libraries are assembled in four steps, beginning with the annealing of 5’ and 3’ oligos (Step 1), followed by extension of the 5’ oligo 3’-end (Step 2), ligation of the produced nick (Step 3) and polymerase chain reaction amplification (Step 4). Oligos used in this study are shown at each step of library assembly with inset gel images showing successful oligo annealing (Step 1) and PCR (Step 4).

**Library Design Considerations:**

Alternative terminator library designs are easily accommodated and may include modification of the flanking (cloning) sequences, the poly-A/T regions, hairpin length and composition so long as they comply with the following design rules:

1. The 5’ and 3’ oligos must base pair in their poly-A/T regions until and including the closing base pairs of the terminator hairpin. Closing the hairpin with one or more G/C pairs and allowing the 3’ oligo to pair to this position is desirable to stabilize oligo binding as is having a flanking region of pairing beyond the poly-A/T stretch. For our library, we chose to use a BamHI restriction cut site for added utility with cloning, however, this sequence may be modified as desired so long as pairing is maintained. Alternative poly-A/T sequences are also tolerable so long as compensatory changes are made to maintain base pairing.
2. The length and composition of the randomized hairpin pair region and loop sequence can be modified so long as the 5’ oligo is capable of folding back on itself to allow self-priming for oligo extension (Step 2).
3. The 3’ oligo must be 5’ monophosphorylated to allow nick ligation at Step 3.
4. The regions flanking the terminator must be known for PCR amplification.

**Supplementary Note 2 – Rationale for Extended Cell Incubation After Induction and Before Flow Cytometry Analysis**

In our preliminary flow cytometry experiments, we discovered that the mBeRFP reporter protein maturation time is approximately 4 hours *in vivo* in *E. coli* and at 37°C, however the fluorescent signal for mBeRFP continues to increase (albeit more slowly) well past 6 hours post-induction. In contrast, the eGFP protein has a much faster maturation time (< 2 hours under the same conditions *in vivo* in *E. coli*). The difference in maturation time between the two proteins results in erroneous measurements in TE until protein maturation for both proteins is complete. We found we had the most reliable measurements for our two fluorescent reporter system after an overnight (16 hour) expression period. Because the cells are induced during mid-log phase and fluorescent proteins have exceptional stability *in vivo*, we interpret that the overall signal for *E. coli* cells in stationary phase (at 16 hours) reflects fluorescent proteins produced during log-phase of growth and that fluorescent protein expression during stationary phase does not substantially change the amount or ratio of either reporter.
