# Supplementary material for: Rapid generation of sequence-diverse terminator libraries and their parameterization using quantitative Term-Seq
Source: Synth Biol (Oxf). 2019 Oct 29;4(1):ysz026. doi: 10.1093/synbio/ysz026 (PMC7445774; doi:10.1093/synbio/ysz026)
Supplement: ysz026_Supplementary_Data [file ysz026_supplementary_data.zip › Supplemental Table 1 - Oligonucleotides used in this study.pdf]

Supplementary Table 1 - Oligonucleotides used in this Study

Primer ID	Sequence (5' to 3')	Comment(s)
oTerm13	GAA TTC GCG GCC GCT TCT AGA GGA TCC **AAA AAA AAS SNN NNN NCA GTG CGA AAG CAC TG**	5' oligo used for terminator library assembly. Underlined sequence is the BioBrick 10 prefix and sequence in bold contains the first part of the terminator including the poly-A stretch, the hairpin 5' arm (red), loop region (black) and first six nt of the 3' arm (blue). S is a G/C and N is any nucleotide.
oTerm14	PO4-**SST TTT TTT T**GG ATC CTA CTA GTA GCG GCC GCT GCA G	3' oligo used for terminator library assembly. The 5' end of the oligo is monophosphorylated and has the last two strong (SS) nucleotides of the terminator hairpin followed by the poly-T tail. The BioBrick 10 suffix sequence is underlined.
oTerm16	TCCCTAGCAAACTGGGGCACAAATAAGACAGAATTCGCGGCCGCTTCTAGAG	Forward primer for PCR amplificaiton of terminator library.
oTerm17	CATATGGTTGTCTCCTTATTGATTTTAGCACTGCAGCGGCCGCTACTAGTA	Reverse primer for PCR amplification of terminator library.
oTermSeq-R	CTCCTCGCCCTTGCTCAC	Antisense oligo used for RNase H digestion of read through pBeRG bicistronic transcripts.
oTerm-Seq-F5-P5	AATGATACGGCGACCACCGAGATCTACACTGGCTGTGGCCAGATACTGCGACCTCCCTAG	Forward oligo for PCR amplificaiton of TermSeq library first-strand cDNAs.
P7-reverse	CAAGCAGAAGACGGCATACGA	Reverse oligo for PCR amplificaiton of TermSeq library first-strand cDNAs.
